# Supplementary material for: Heat-activated growth of metastable and length-defined DNA fibers expands traditional polymer assembly
Source: Nat Commun. 2024 May 23;15:4384. doi: 10.1038/s41467-024-48722-2 (PMC11116425; doi:10.1038/s41467-024-48722-2)
Supplement: Supplementary file 1 — Supplementary Information [file 41467_2024_48722_MOESM1_ESM.pdf]

Supplementary Information

Heat-Activated Growth of Metastable and Length-Defined DNA Fibers Expands Traditional Polymer Assembly

Michael D. Dore[1§], Muhammad Ghufran Rafique[1§], Tianxiao Peter Yang[2], Marlo Zorman[3], Casey M. Platnich[1], Pengfei Xu[1], Tuan Trinh[1], Felix J. Rizzuto[4], Gonzalo Cosa[1,5], Jianing Li[6], Alba Guarné[2,5], and Hanadi F. Sleiman[1,5*]

[1]Department of Chemistry, McGill University, 801 Sherbrooke St W, Montréal, QC H3A 08B, Canada
[2]Department of Biochemistry and Centre de Recherche en Biologie Structurale, McGill University, Montréal, Québec, Canada.
[3]Department of Chemistry, University of Vermont, Burlington, VT 05405, USA
[4]School of Chemistry, University of New South Wales, Sydney, 2052, Australia
[5]Centre de Recherche en Biologie Structurale, McGill University, Montréal, Québec, Canada.
[6]Department of Medicinal Chemistry and Molecular Pharmacology, Purdue University, West Lafayette, IN 47906, USA
[§]These authors contributed equally
* hanadi.sleiman@mcgill.ca

Table of Contents

SI-I. Supplementary Items 3

SI-II. Supplementary Experimental Procedures 25

II-a. General materials and DNA-oligomer sequences 25

II-b. Instrumentation 25

II-c. Synthesis of branched alkyl chain phosphoramidites 26

Branched C12 phosphoramidite 26

Branched C8 phosphoramidite 26

II-d. Solid-phase synthesis and purification of sequence-defined DNA-oligomers 28

Solid-phase synthesis of branched DNA-oligomers 28

Purification of branched DNA-oligomers 28

II-e. Dynamic light scattering 32

II-f. Variable temperature assembly studies 32

II-g. Estimation of molecular geometry 32

II-h. Molecular dynamics simulations 32

II-i. Seeded growth of length defined fibers 32

II-j. Förster resonance energy transfer (FRET) studies 33

References 34

SI-I. Supplementary Items

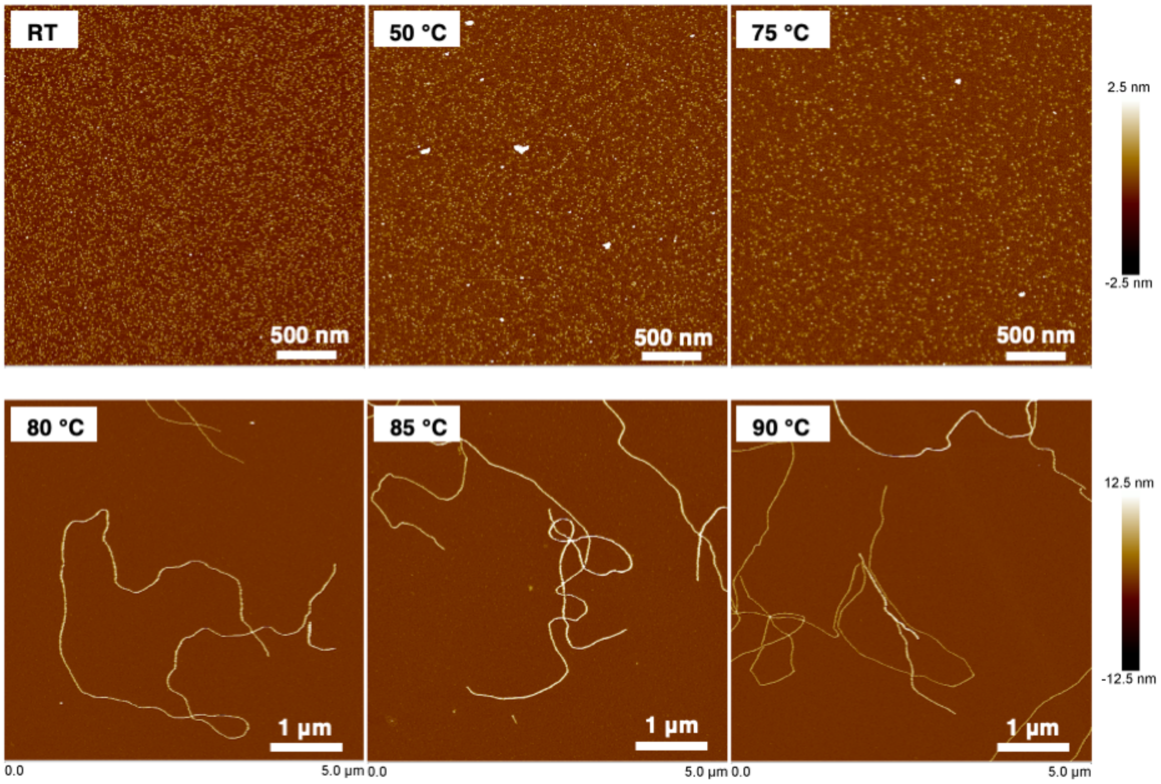

Supplementary Figure 1| bC8$_8$-DNAa at temperatures below and above the assembly temperature, heated at a different rate compared to Supplementary Fig. 2 and 3. Samples were heated from 20 to 90 °C incrementally by holding for 10 minutes at 30, 40, 50, 60, 70, 75, 80, 85 and 90 °C. Aliquots were deposited on mica at indicated temperatures then immediately washed and dried. AFM in air on mica. 7.5 µM DNA-oligomer in 6.25 mM Mg^{2+}.

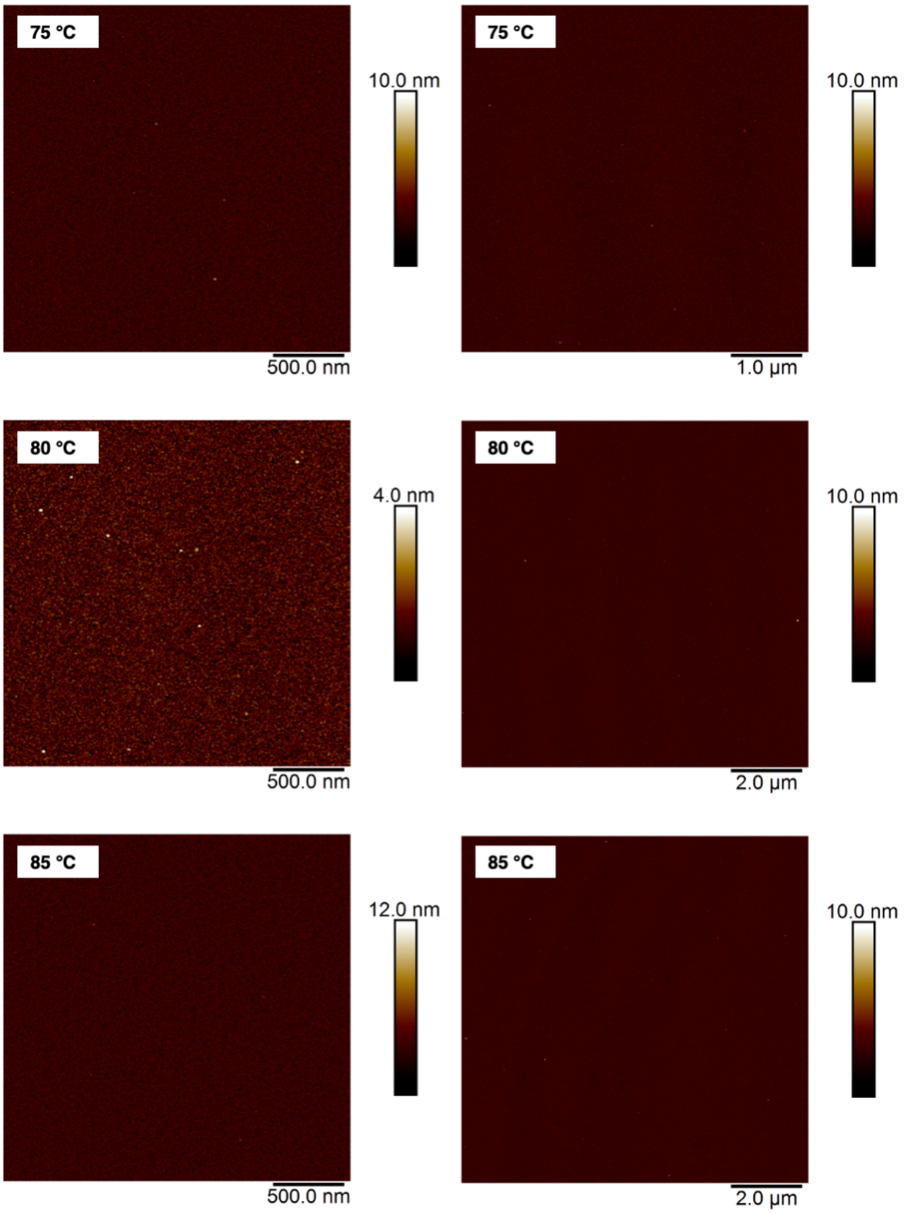

Supplementary Figure 2| bC8$_8$-DNAa at temperatures below assembly. Samples were heated from 25 to 99 °C at 0.5 °C per minute. Aliquots were deposited on mica at indicated temperatures then immediately washed and dried. AFM in air on mica. 7.5 μM DNA-oligomer in 6.25 mM Mg^{2+}.

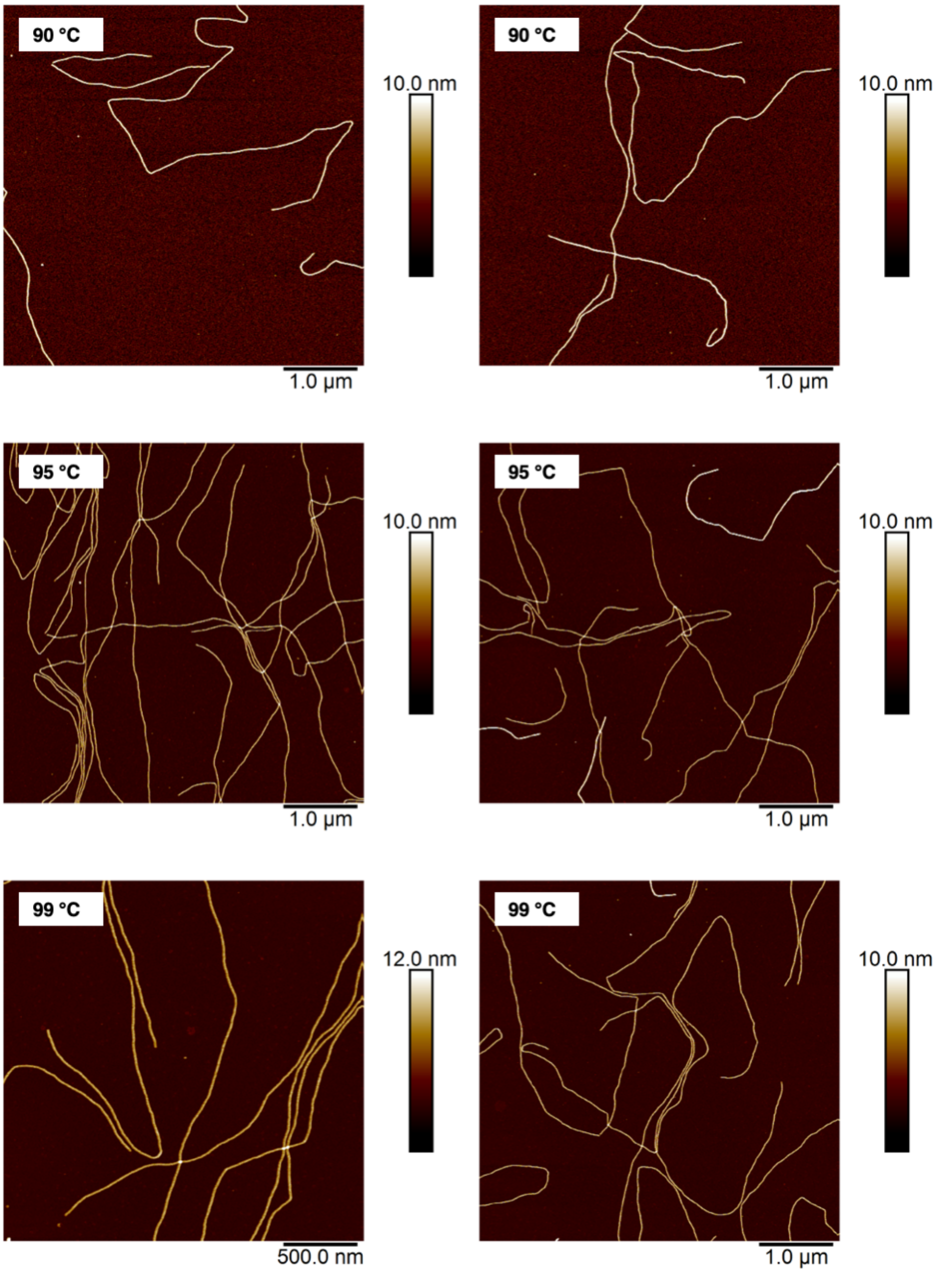

Supplementary Figure 3| bC8$_8$-DNAa at temperatures above assembly. Samples were heated from 25 to 99 °C at 0.5 °C per minute. Aliquots were deposited on mica at indicated temperatures then immediately washed and dried. AFM in air on mica. 7.5 µM DNA-oligomer in 6.25 mM Mg^{2+}.

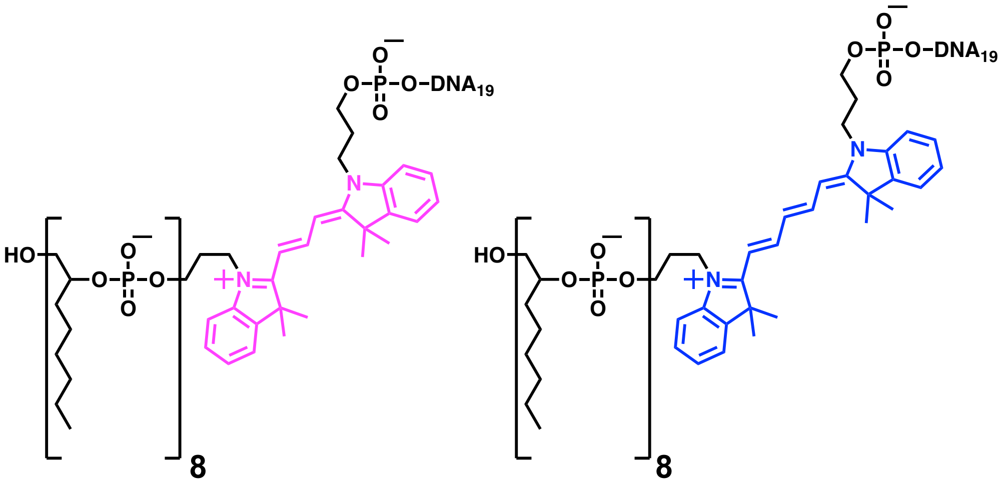

Supplementary Figure 4| Molecular structure of bC8$_8$-Cy3-DNAa (left) and bC8$_8$-Cy5-DNAa (right).

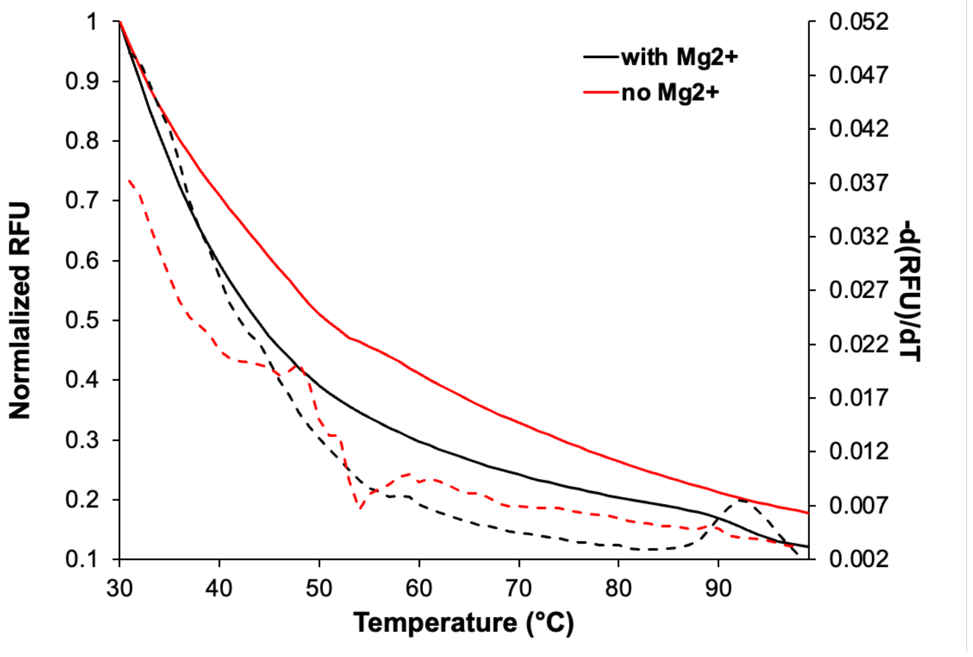

Supplementary Figure 5| Normalized fluorescence and derivative of fluorescence (dashed line) of bC8$_8$-Cy3-DNAa with 6.25 mM Mg^{2+} or water as temperature was increased from 25 to 99 °C at 0.5 °C per minute. Detailed view of 80 to 99 °C shown in Fig. 1C. Fluorescence was measure in situ at the specified temperatures.

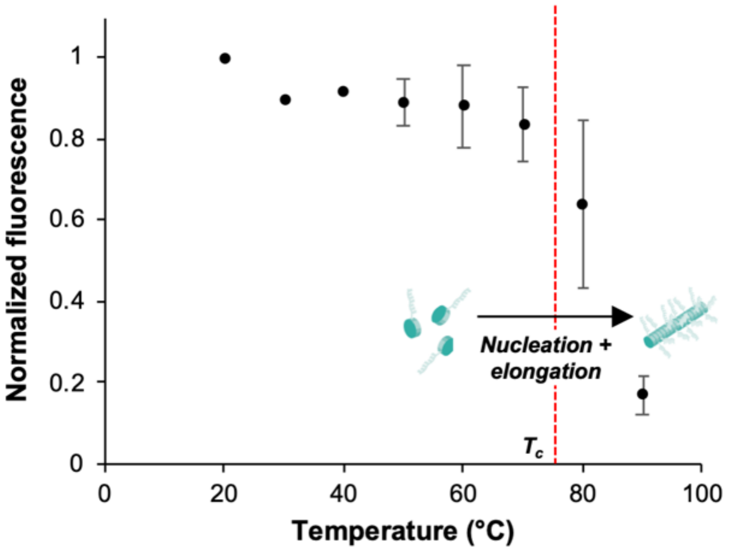

Supplementary Figure 6| bC8$_8$-Cy3-DNAa fluorescence at temperatures below and above the assembly temperature, heated at the same rate as Supplementary Fig. 1. Samples were heated from 20 to 90 °C incrementally by holding for 10 minutes at 30, 40, 50, 60, 70, 75, 80, 85 and 90 °C. Aliquots were removed at each time point, immeediately cooled to room temperature, and the fluorescence measured on a plate reader. Excitation: 530 nm, emission: 570 nm. 7.5 μM DNA-oligomer in 6.25 mM Mg^{2+}.

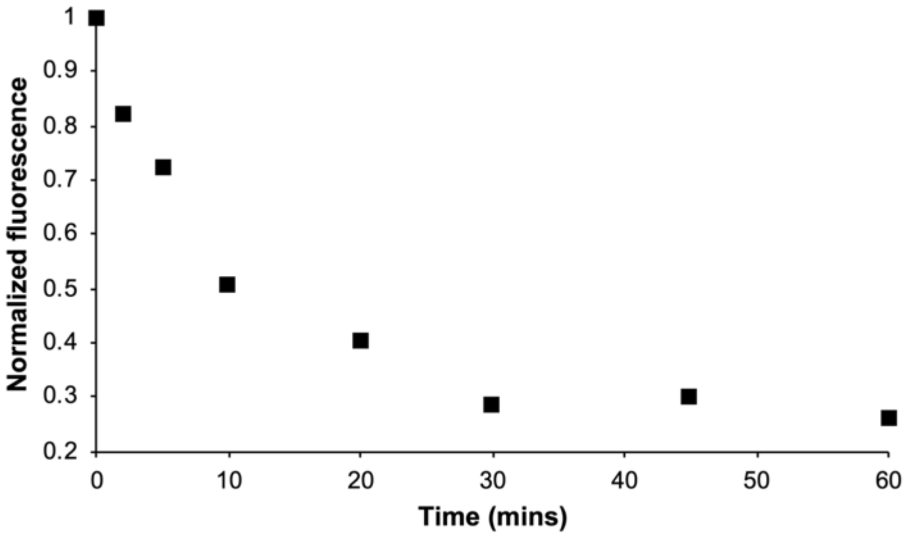

Supplementary Figure 7| Normalized fluorescence of bC8$_8$-Cy3-DNAa following heating at 90 °C for different lengths of time. Aliquots were cooled to room temperature before measuring fluorescence on a plate reader.

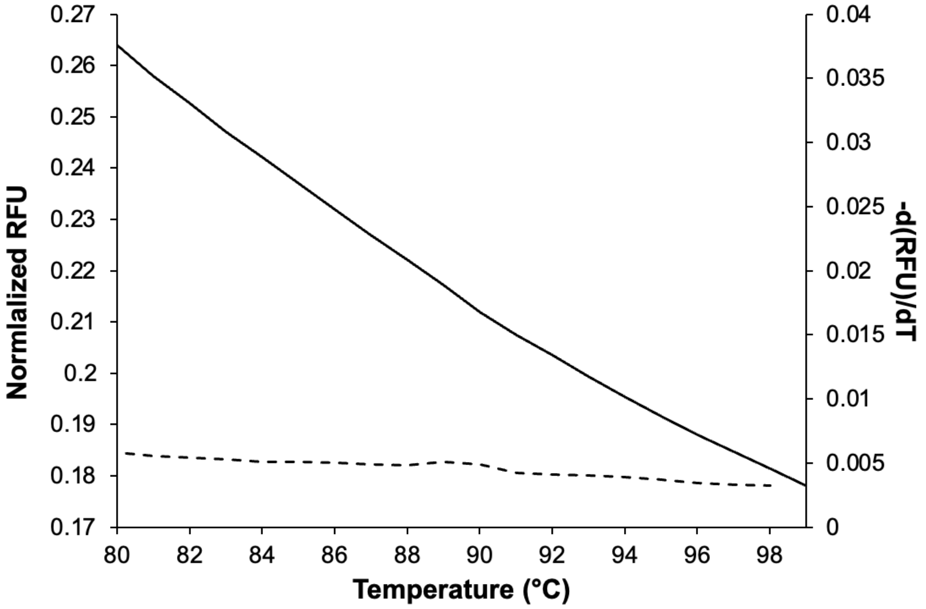

Supplementary Figure 8| Normalized fluorescence and derivative of fluorescence of $bC8_8$-Cy3-DNAa in water as temperature was increased from 25 to 99 °C at 0.5 °C per minute. Detailed view of part of Supplementary Fig. 5. Fluorescence was measure in situ at the specified temperatures.

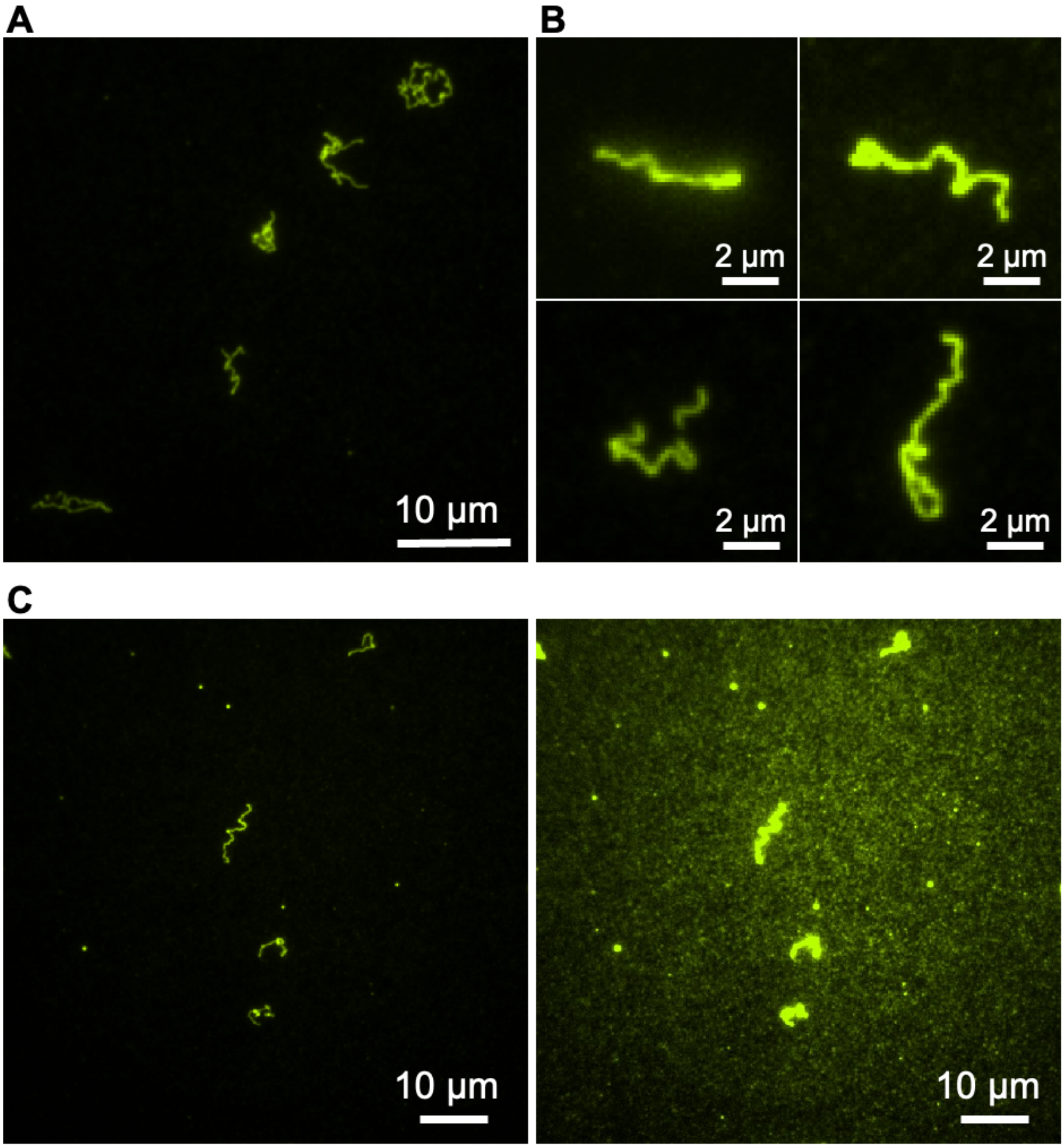

Supplementary Figure 9| Total internal reflection fluorescence microscopy images of 9 µM of bC8$_8$-DNAa and 1 µM of bC8$_8$-Cy3-DNAa mixed in 0.5 X TAMg (6.25 mM Mg^{2+}) then heated at a rate of 1 °C per minute to 75 °C and held for 6 hours before cooling to room temperature. Surface was functionalized with a complementary DNA strand. **A.** Additional image. **B.** Additional close-up images of individual fibers. **C.** Demonstration of low background fluorescence. Image on right has increased brightness and lowered contrast. Imaging performed at low power (0.7 mW as measured from the objective) and low gain, to not saturate the pixels. As a result, the fibers are much brighter than the background in these imaging conditions that single fluorophores that are non-specifically attached to the surface are not visible.

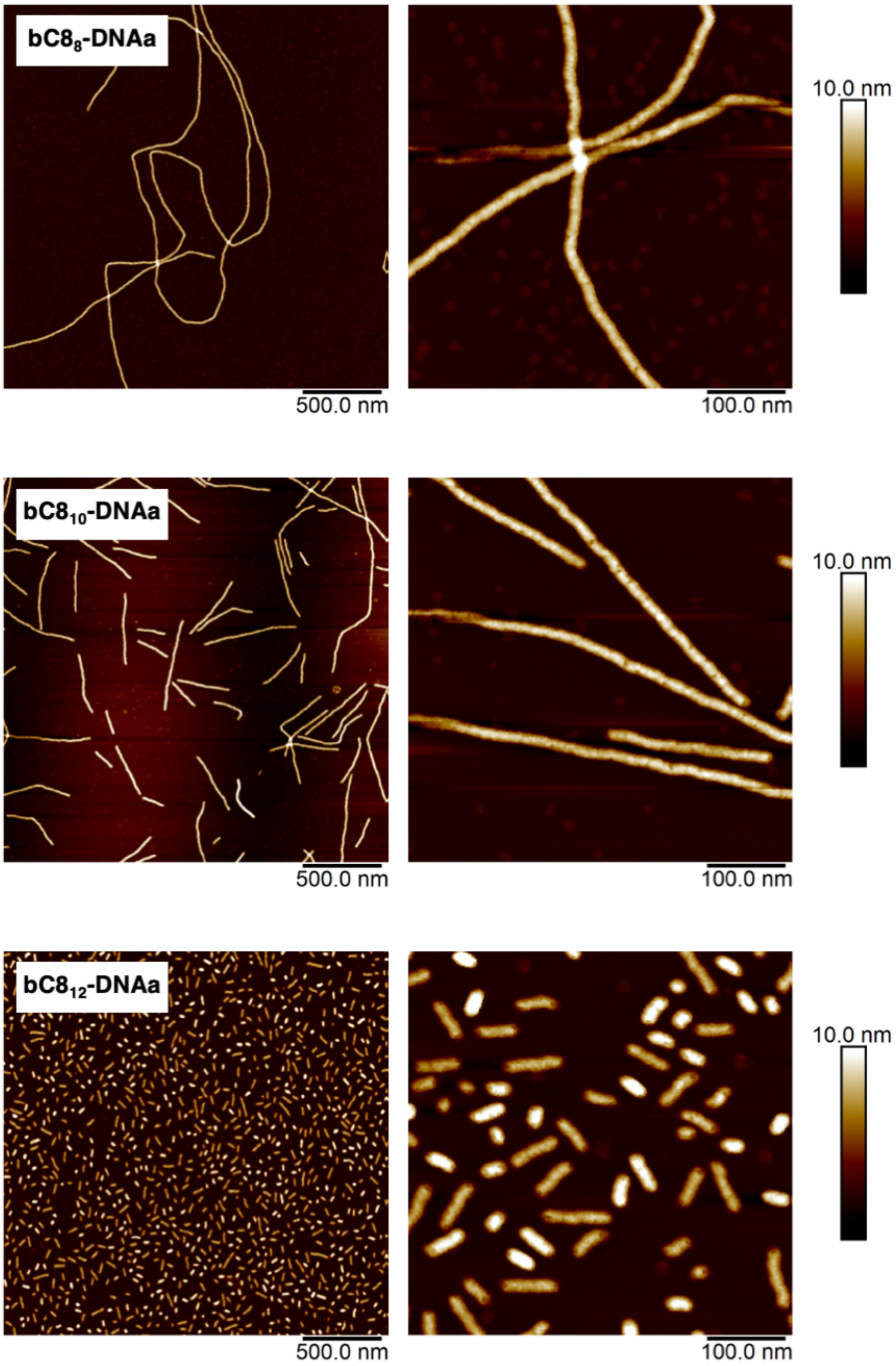

Supplementary Figure 10| DNA oligomers heated at 0.5 °C per minute from 25 to 99 °C, then quickly cooled to room temperature. AFM in air on mica. 7.5 μM DNA-oligomer in 6.25 mM Mg^{2+}.

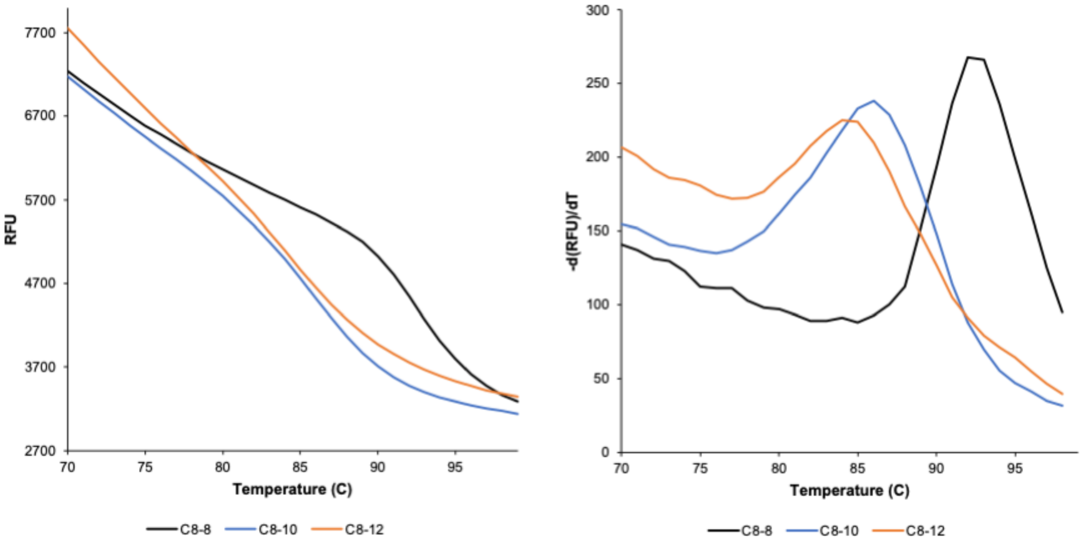

Supplementary Figure 11| Fluorescence vs temperature curves of DNA oligomers heated at 0.5 °C / min from 25 to 99 °C. 7.5 μM DNA-oligomer in 6.25 mM Mg^{2+}. C8-8 corresponds to $bC8_8$-Cy3-DNAa, C8-10 to $bC8_{10}$-Cy3-DNAa and C8-12 to $bC8_{12}$-Cy3-DNAa. C8-8 data is reproduced from Figure 1 as comparison. Fluorescence measurements showed self-assembly transitions at 86 °C for $bC8_{10}$-Cy3-DNAa and 84 °C for $bC8_{12}$-Cy3-DNAa, compared to 92 °C for $bC8_8$-Cy3-DNAa. This trend suggested a higher propensity for assembly as the number of bC8 monomers increased, despite the observed decrease in fiber length. The lower assembly temperature for $bC8_{10}$-Cy3-DNAa and $bC8_{12}$-Cy3-DNAa could be due to their higher hydrophobicity; however, the reduced lengths of the resultant fibers signal less efficient molecular packing as hydrophobicity increased. Fluorescence was measure in situ at the specified temperatures.

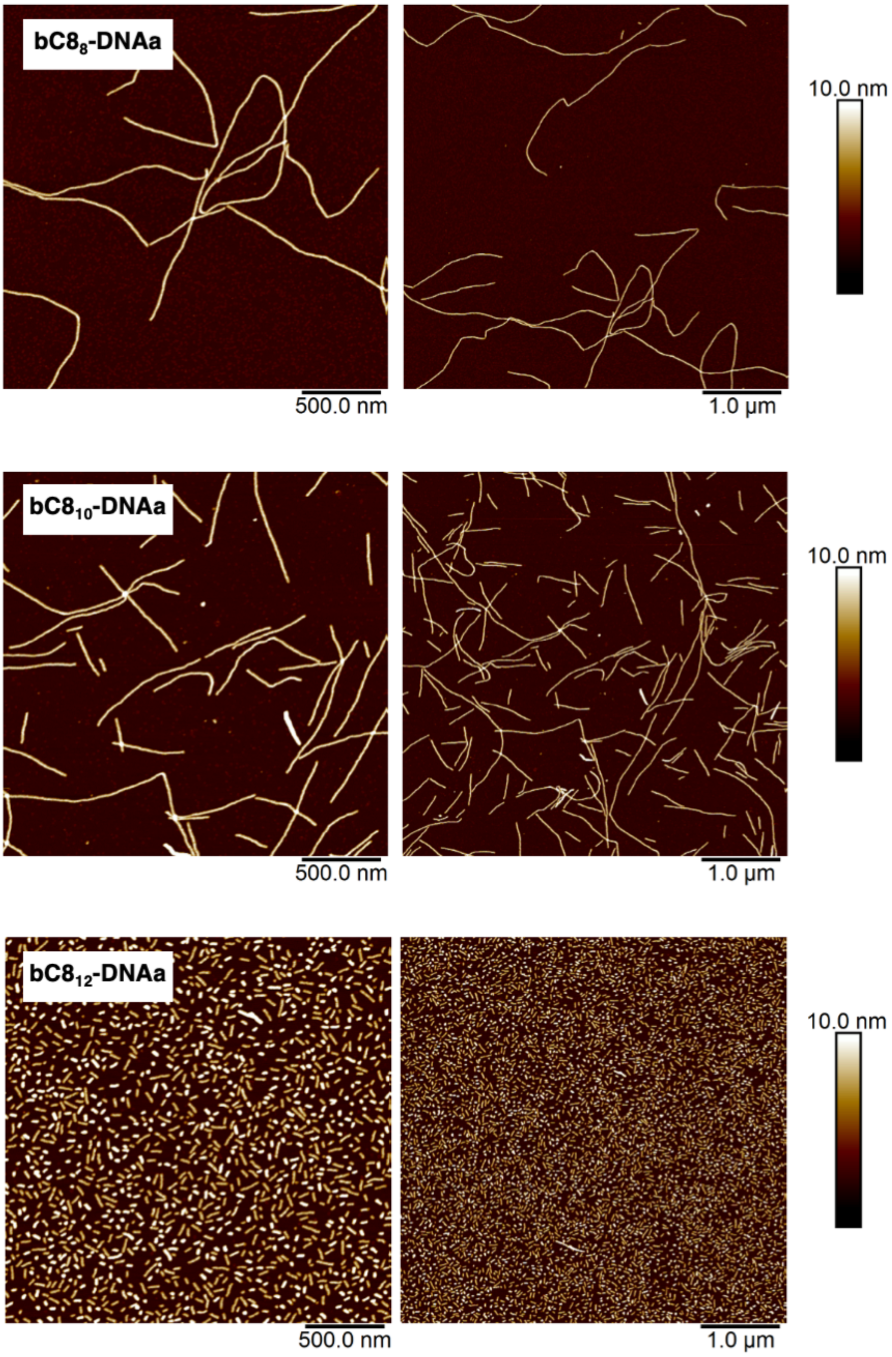

Supplementary Figure 12| DNA oligomers heated at 1 °C per minute from 25 to 99 °C, then cooled to room temperature at 1 °C per minute. AFM in air on mica. 7.5 µM DNA-oligomer in 6.25 mM Mg^{2+}.

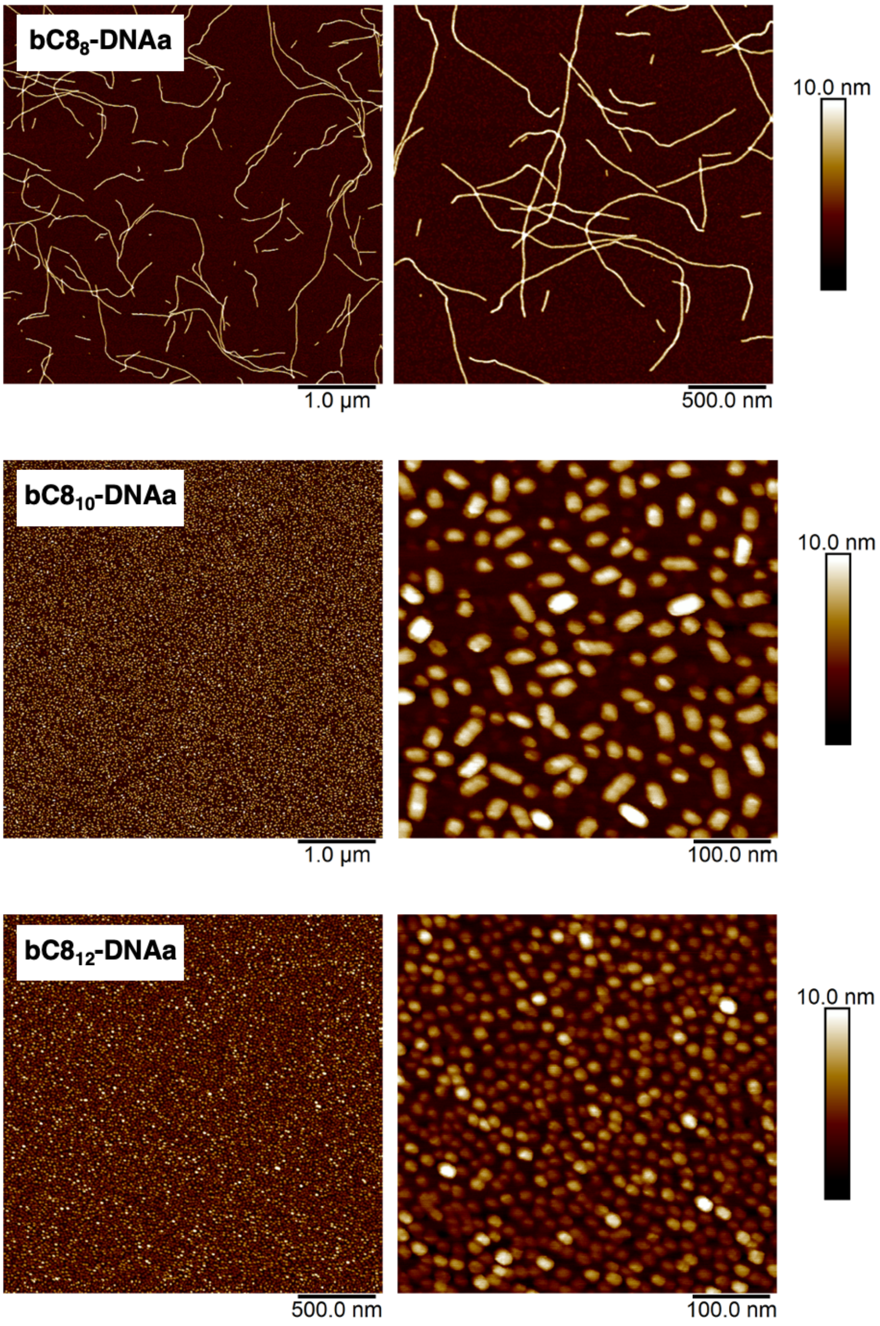

Supplementary Figure 13| DNA oligomers directly heated at 99 °C for 10 minutes then cooled at 1 °C / min to room temperature. AFM in air on mica. 7.5 µM DNA-oligomer in 6.25 mM Mg^{2+}.

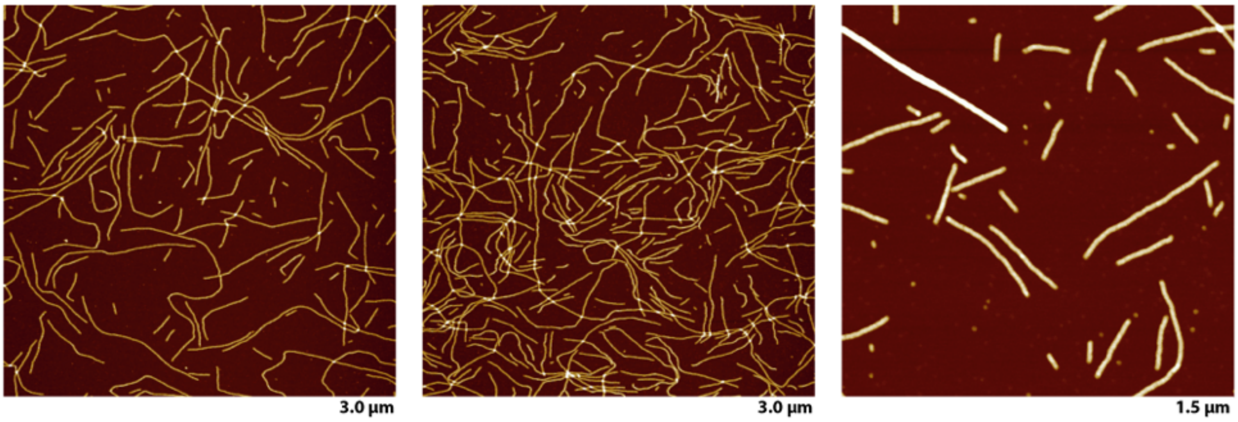

Supplementary Figure 14| Additional AFM images in air on mica of C8$_8$-DNAa at 10 µM. [Mg^{2+}] = 12.5 mM. Height scale: 15 nm (first two images), 12.5 nm (far right image). DNA oligomers were directly heated at 99 °C for 10 minutes then cooled at 1 °C / min to room temperature.

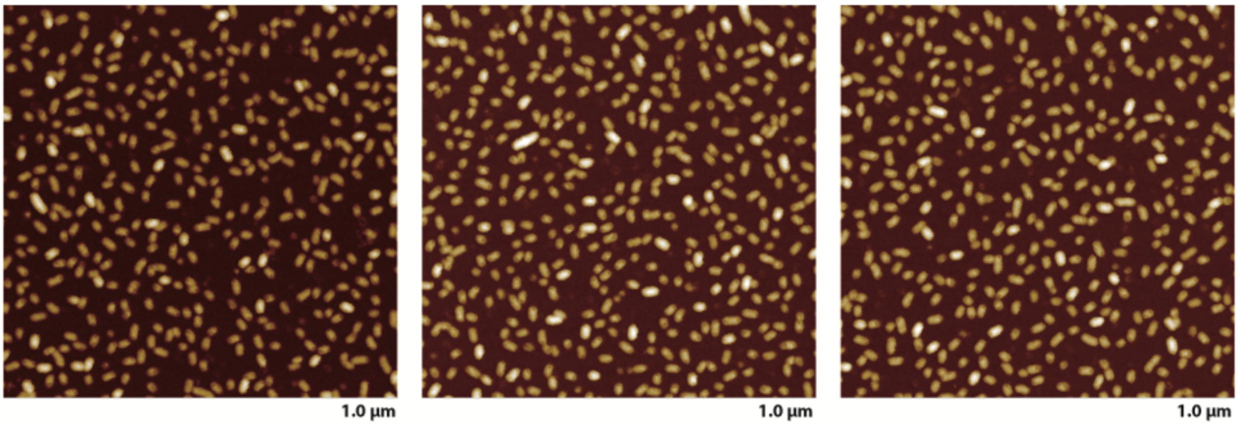

Supplementary Figure 15| Additional AFM images in air on mica of C8$_{10}$-DNAa at 10 µM. [Mg^{2+}] = 12.5 mM. Height scale: 15 nm. DNA oligomers were directly heated at 99 °C for 10 minutes then cooled at 1 °C / min to room temperature.

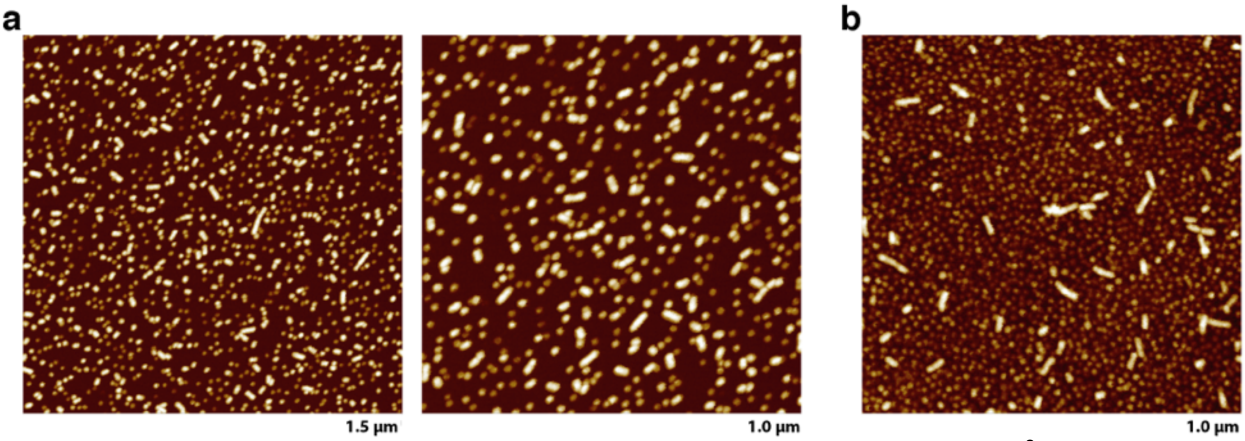

Supplementary Figure 16| Additional AFM images in air of C8$_{12}$-DNAa at [Mg^{2+}] = 12.5 mM, height scale: 15 nm. **a**. At 10 µM on mica. **b**. At 7.5 µM on HOPG. DNA oligomers were directly heated at 99 °C for 10 minutes then cooled at 1 °C / min to room temperature. Distances underneath images correspond to total width of the micrograph.

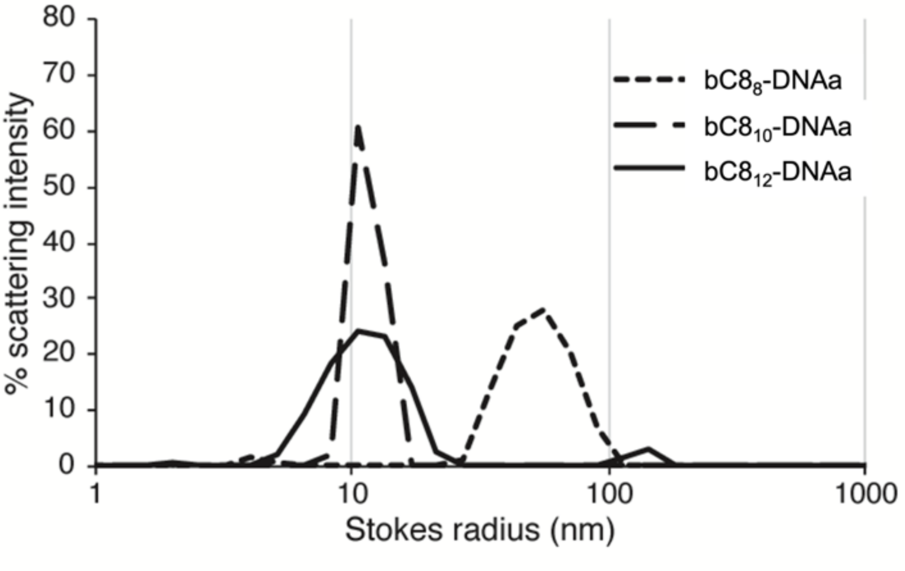

Supplementary Figure 17| Dynamic light scattering intensity vs. radius for bC8 containing branched DNA-oligomers assuming a spherical morphology. In 6.25 mM Mg^{2+} at 10 μM DNA. The determined Stokes radius doesn't directly apply to fibre morphologies, but a large size difference between bC8$_{12}$-DNAa and bC8$_8$-DNAa is evident, as observed by AFM. NA oligomers were directly heated at 99 °C for 10 minutes then cooled at 1 °C / min to room temperature

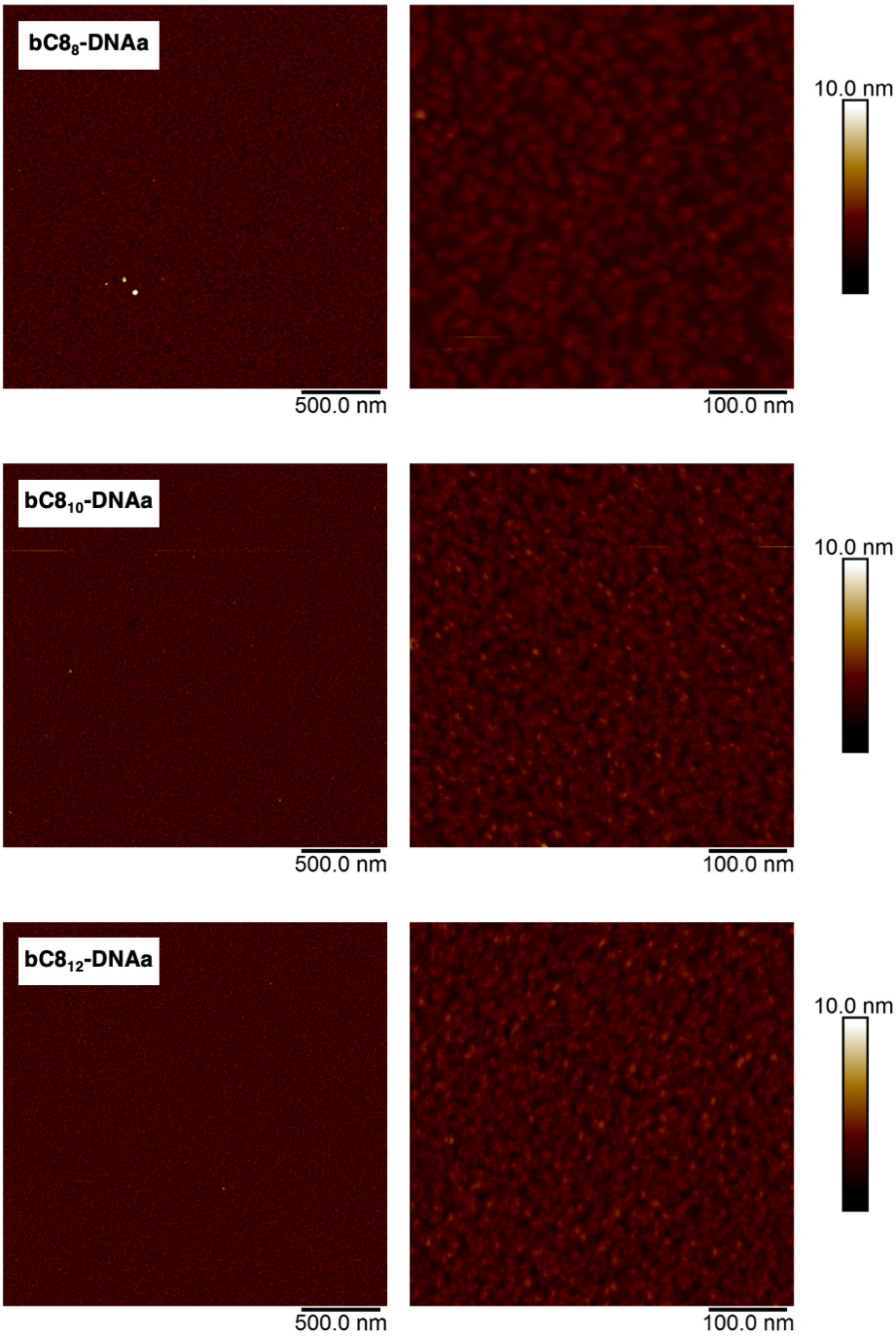

Supplementary Figure 18| DNA oligomers at room temperature prior to any heating. AFM in air on mica. 7.5 µM DNA-oligomer in 6.25 mM Mg^{2+}.

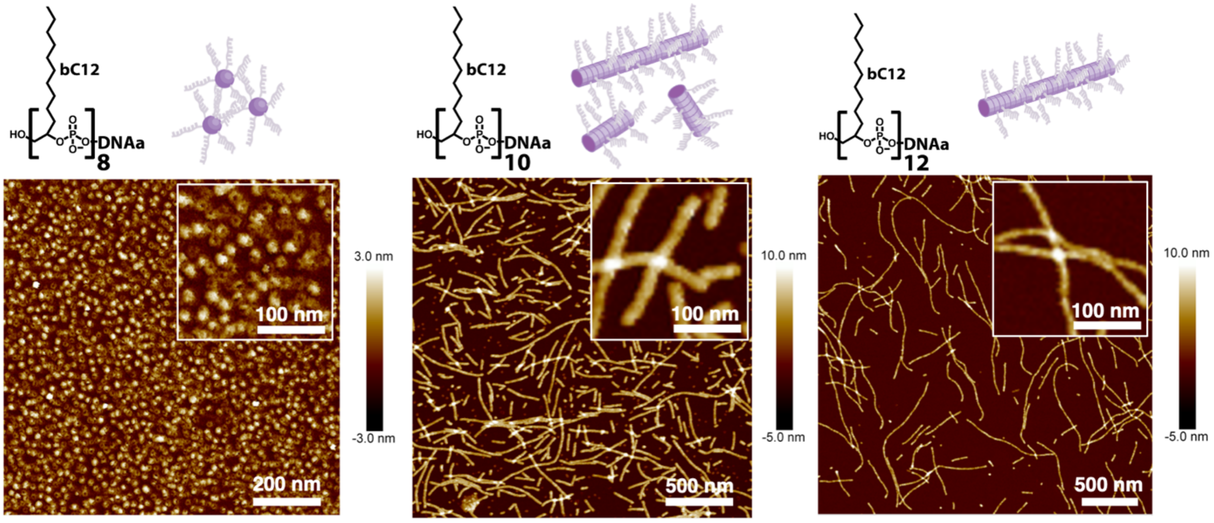

Supplementary Figure 19| Appending increasing numbers of bC12 to DNA results in the opposite trend in morphology (from spheres to fibers) compared to bC8. AFM images in air on mica. Samples assembled by heating rapidly to 95 °C and cooling 1 °C / min to room temperature. In 12.5 mM Mg^{2+} at 10 µM DNA

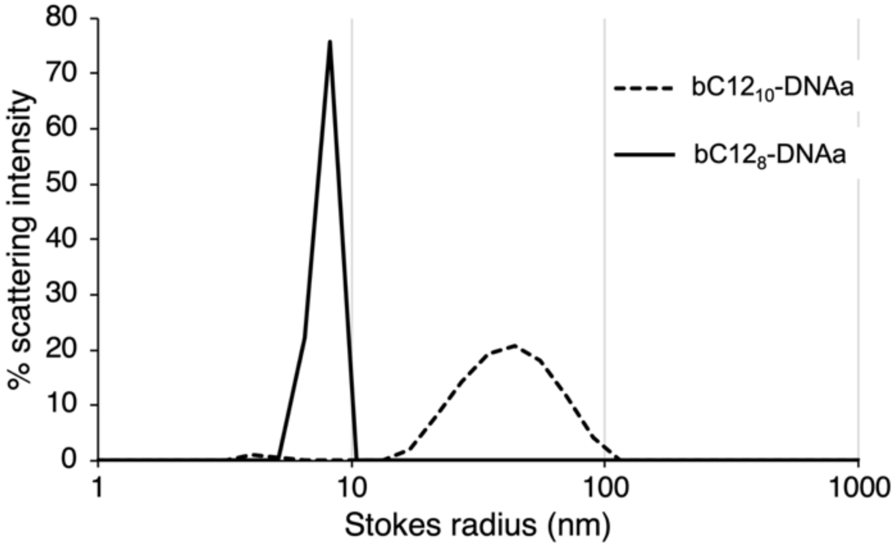

Supplementary Figure 20| Dynamic light scattering intensity vs. radius for bC12 containing branched DNA-oligomers assuming a spherical morphology. In 12.5 mM Mg^{2+} at 10 µM DNA. The determined Stokes radius doesn't directly apply to fibre morphologies, but a large size difference between bC12$_{10}$-DNAa and bC12$_{8}$-DNAa is evident, as observed by AFM.

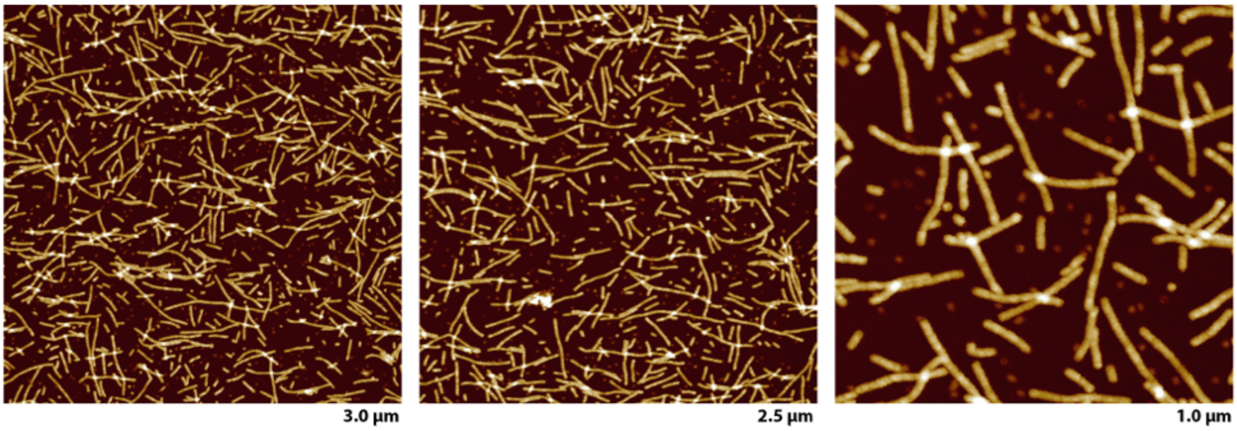

Supplementary Figure 21| Additional AFM images in air on mica of C12$_{10}$-DNAa at 10 µM. [Mg^{2+}] = 12.5 mM. Height scale: 17.5 nm. Distances underneath images correspond to total width of the micrograph.

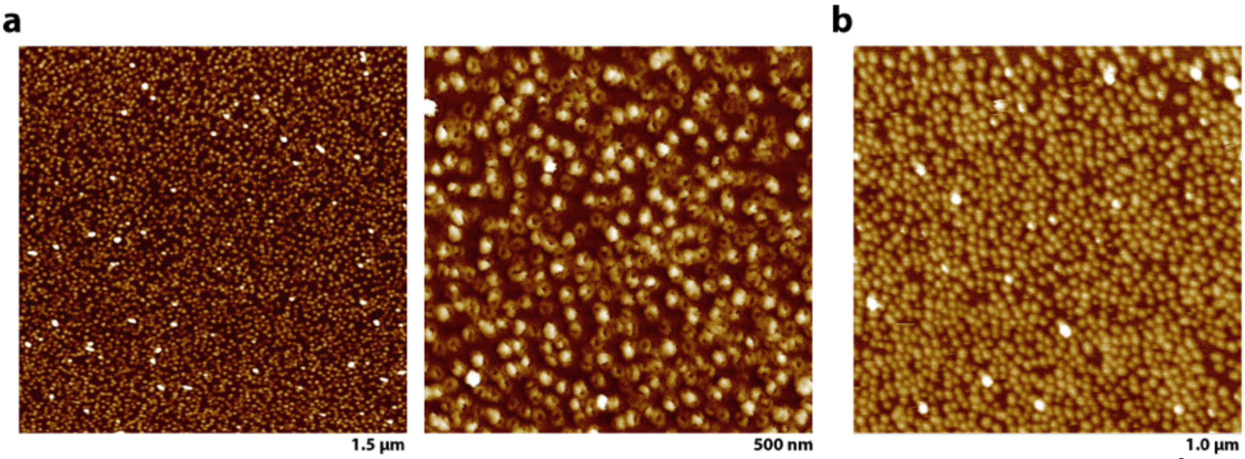

Supplementary Figure 22| Additional AFM images on mica of C12$_8$-DNAa at 10 µM. [Mg^{2+}] = 12.5 mM. **a**. In air, height scale: 6 nm. **b**. In liquid (1 X TAMg), height scale: 10 nm. Distances underneath images correspond to total width of the micrograph.

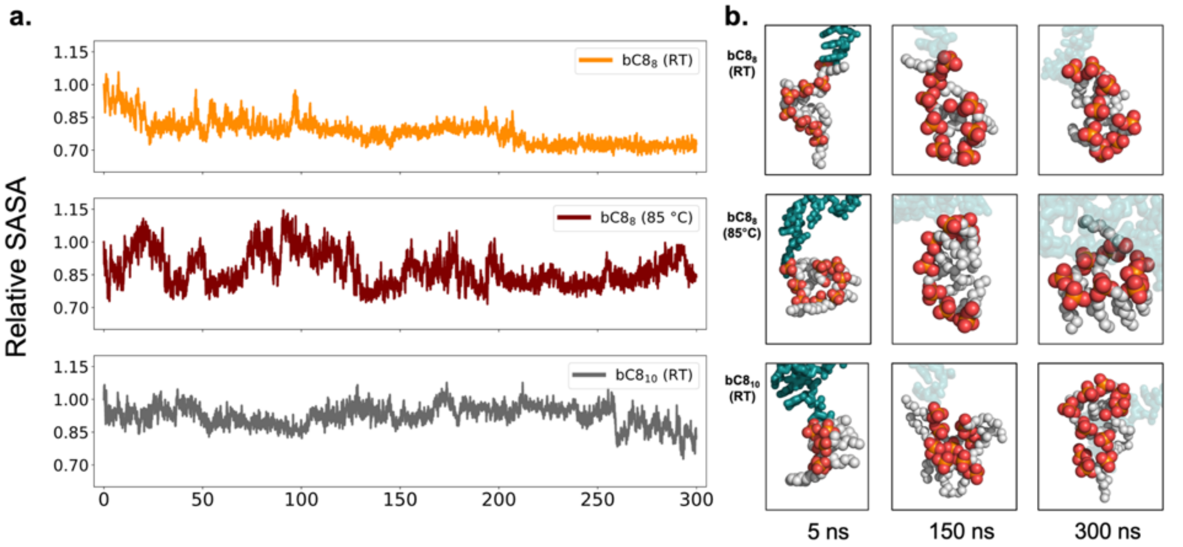

Supplementary Figure 23| Simulations of bC8$_8$-DNAa and bC8$_{10}$-DNAa reveal differences in oligomer folding. a. Relative SASA calculations of hydrophobic blocks show that bC8$_8$-DNAa folds into a stable conformation at room temperature and interconverts between folded and unfolded states at 85 °C, whereas bC8$_{10}$-DNAa consistently remains unfolded. **b.** Snapshots of bC8$_8$-DNAa and bC8$_{10}$-DNAa showing folded and unfolded conformations.

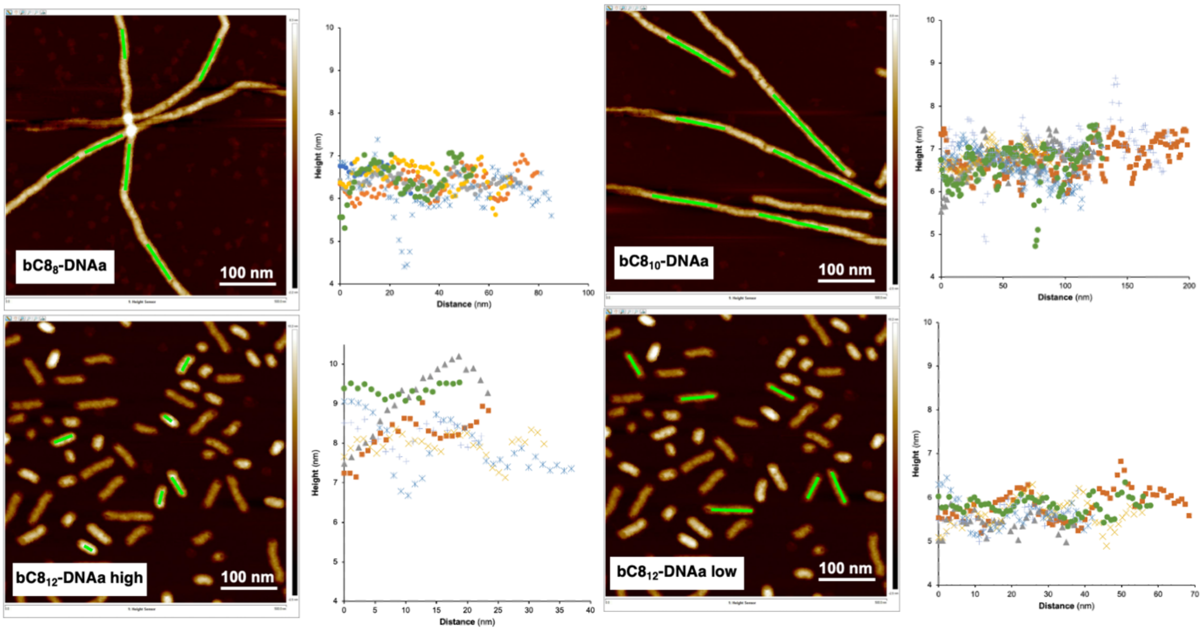

Supplementary Figure 24| Representative AFM images used to measure height of nanostructures. Green lines indicate measurement sections. Nanostructures were assembled by heating slowly (1°C / min) to 99 °C and cooling quickly.

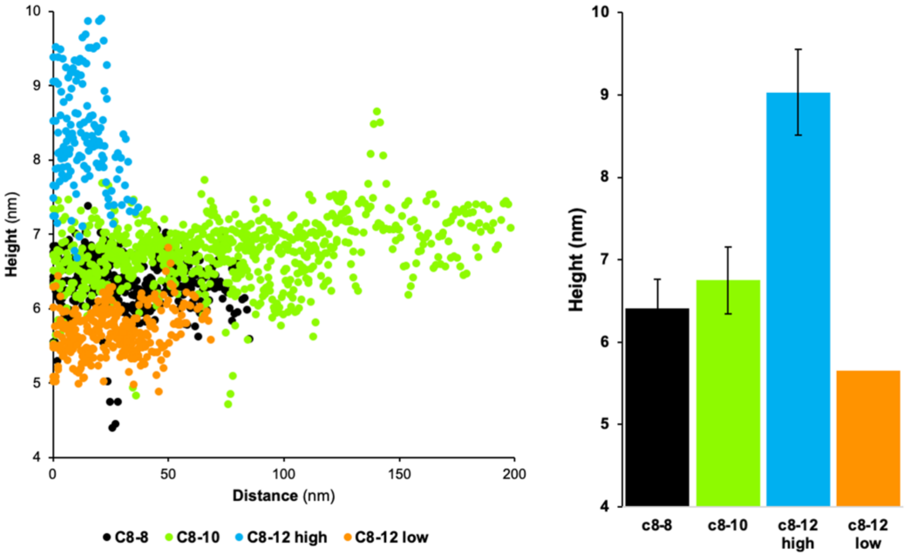

Supplementary Figure 25| Comparison of height statistics from measurements detailed in Supplementary Fig. 22. For bC8$_{12}$-DNAa, two populations with different heights were observed: short fibers with a height of 5.66 (± 0.30) nm and very short, higher cylinders at 9.03 (± 0.53) nm. The greater height of the very short cylinders and their inability to elongate into fibers suggests a more dense, globular conformation of the oligomers. The lower height of the other short fibers could indicate a structure in which the branched oligomeric core assumes a more helical conformation to maintain a smaller core diameter, resulting in a lower density of DNA in the corona and hence a lower height by AFM. The short length of these fibers suggests that this conformation is less conducive to forming long fibers compared to bC8$_8$-DNAa.

Supporting Table 1| Summary of AFM characterization data for bC8 DNA-amphiphiles. Assembly protocol: Heat 0.5 °C / min to 99 °C, cool quickly to room temperature (slow heat, fast cool). Measurements were taken along the fibers. Six measurements were taken for each morphology, where N = the total number of data points within those measurements).

Oligomer	Height (nm)	*L*/*N*	Morphology
**$C8_8$-DNAa**	6.41 (± 0.35), N = 372	1	Long fibres
**$C8_{10}$-DNAa**	6.75 (± 0.41), N = 773	0.8	Short fibers
**$C8_{12}$-DNAa**	high: 9.03 (± 0.53), N = 143<br>low: 5.66 (± 0.30), N = 372	0.67	Very short fibers (low) and shorter cylinders (high)

Supporting Table 2| Summary of DLS and AFM characterization data for various branched DNA-amphiphiles. Assembly protocol: Heat directly to 95 °C, cool 1 °C / min to room temperature (fast heat, slow cool). *D*: diffusion coefficient; % Pd: percentage polydispersity. DLS Measurements performed in triplicate. AFM measurements were taken as cross-sections.

Oligomer	*D* (cm^2/s) E-8	% Pd	Height (nm)	Diameter (nm)	*L*/*N*	Morphology
**$C12_{10}$-DNAa**	7.08 (± 1.68)	30.5 (± 11.6)	8.5 (± 0.6), N = 30	27.5 (± 1.8), N = 60	1.2	shorter fibres
**$C12_8$-DNAa**	29.7 (± 0.23)	13.9 (± 4.4)	collapsed	21.4 (± 2.2), N = 54	1.5	spheres
**$C8_8$-DNAa**	4.59 (± 0.30)	29.0 (± 14.3)	6.1 (± 0.3), N = 60	27.2 (± 1.9), N = 60	1	fibres
**$C8_{10}$-DNAa**	21.4 (± 0.11)	14.3 (± 7.7)	6.2 (± 1.1), N = 30	26.4 (± 1.4), N = 60	0.8	short cylinders
**$C8_{12}$-DNAa**	21.0 (± 0.02)	35.0 (± 3.8)	high: 9.5 (± 0.6), N = 30<br>low: 5.8 (± 1.1), N = 30	cylinders: 30.6 (± 3.0), N = 18<br>spheres: 24.1 (± 2.3), N = 60	0.67	short cylinders, spheres

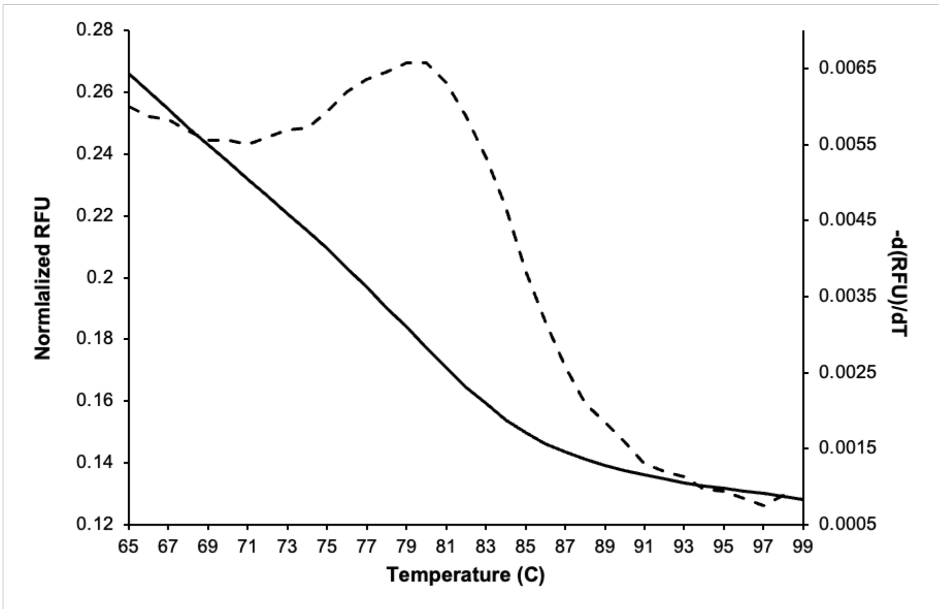

Supplementary Figure 26| Fluorescence vs temperature curves of DNA oligomers heated at 0.5 °C / min from 25 to 99 °C. 7.5 µM bC8$_8$-Cy3-DNAb in 6.25 mM Mg^{2+} with 0.1 molar equivalents of bC8$_{10}$-DNAa seeds.

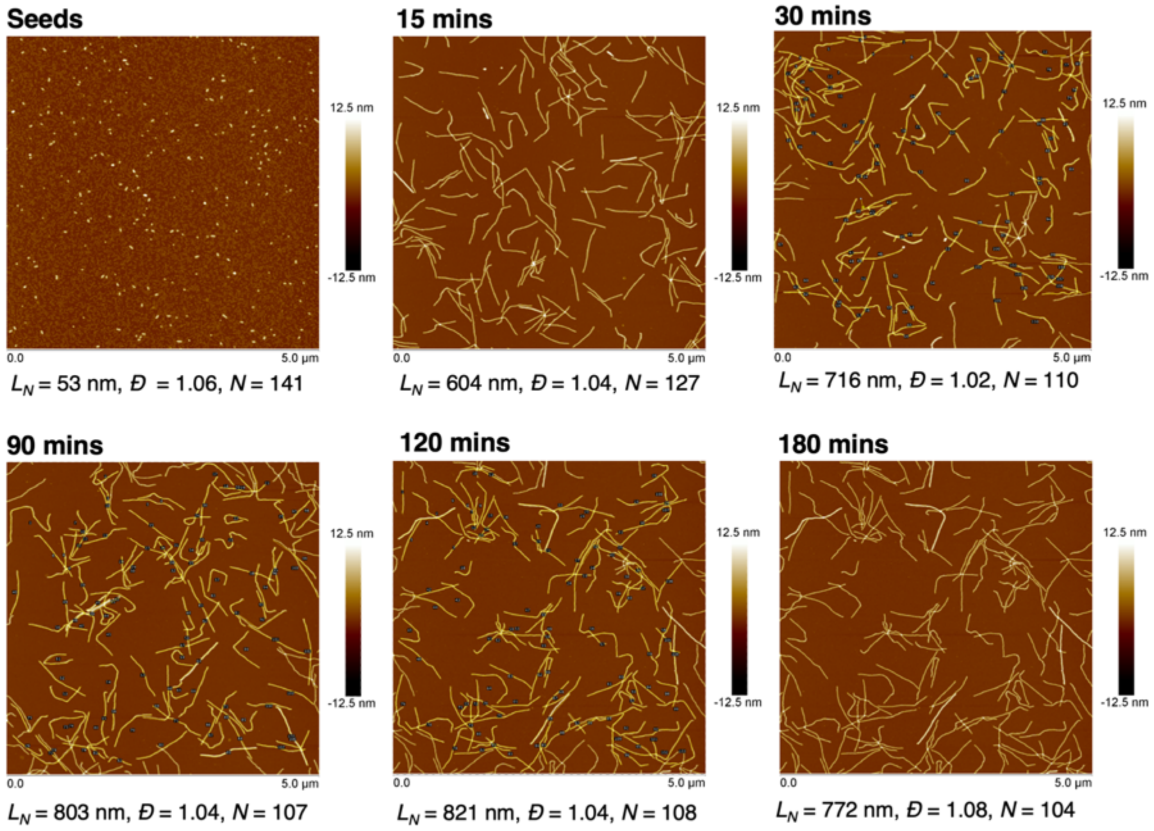

Supplementary Figure 27| Seeded growth of fibers over time with 6.25 mM Mg^{2+}. AFM on mica in air. Molar ratio of bC8$_{10}$-DNAa to bC8$_8$-DNAb of 1 to 20. The differences in L_N for samples 60 (Figure 4B), 90, 120, 180 mins are not statistically significant. Distances underneath images correspond to total width of the micrograph.

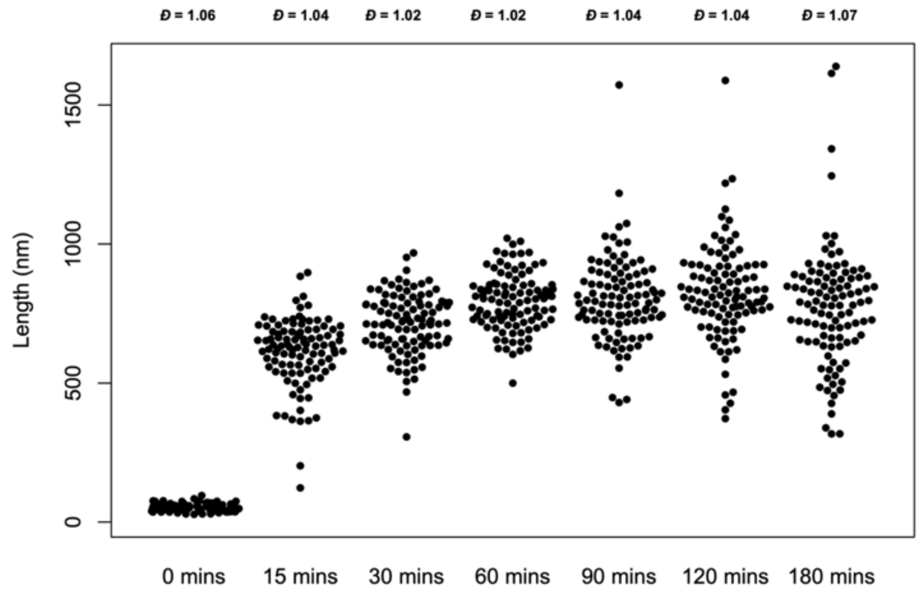

Supplementary Figure 28| Seeded growth of fibers over time with 6.25 mM Mg^{2+}. Length stops increasing at 60 minutes, but dispersity begins to increase after 60 minutes (labelled above).

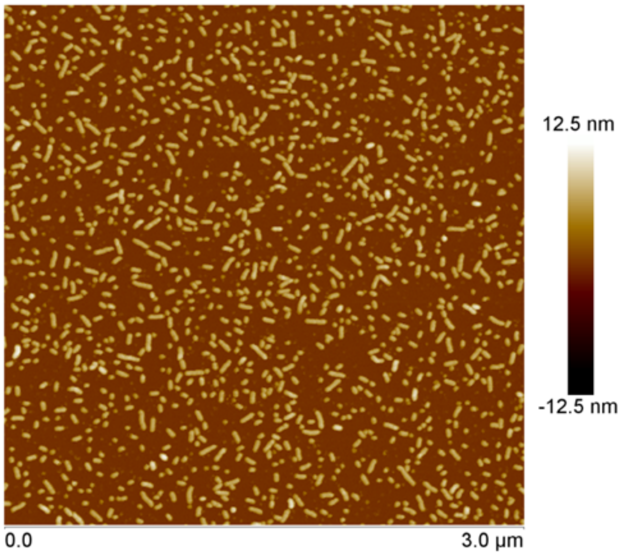

Supplementary Figure 29| $bC8_{10}$-DNAa seed particles following aging at 10 °C for three months. AFM on mica in air. Distances underneath images correspond to total width of the micrograph.

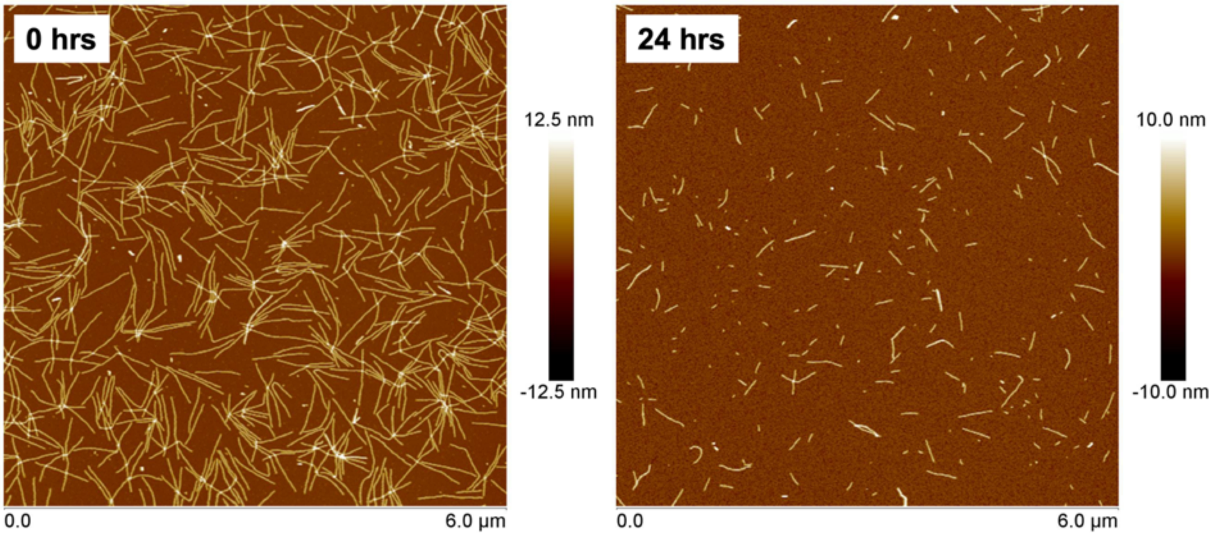

Supplementary Figure 30| Separately assembled bC8$_8$-Cy3-DNAa and bC8$_8$-Cy5-DNAa at 0 hours and 24 hours following mixing. AFM in air on mica. At 0 hours: L_N = 600 nm, D = 1.04 (N = 76); at 24 hours: L_N = 165 nm, D = 1.37 (N = 155). Distances underneath images correspond to total width of the micrograph.

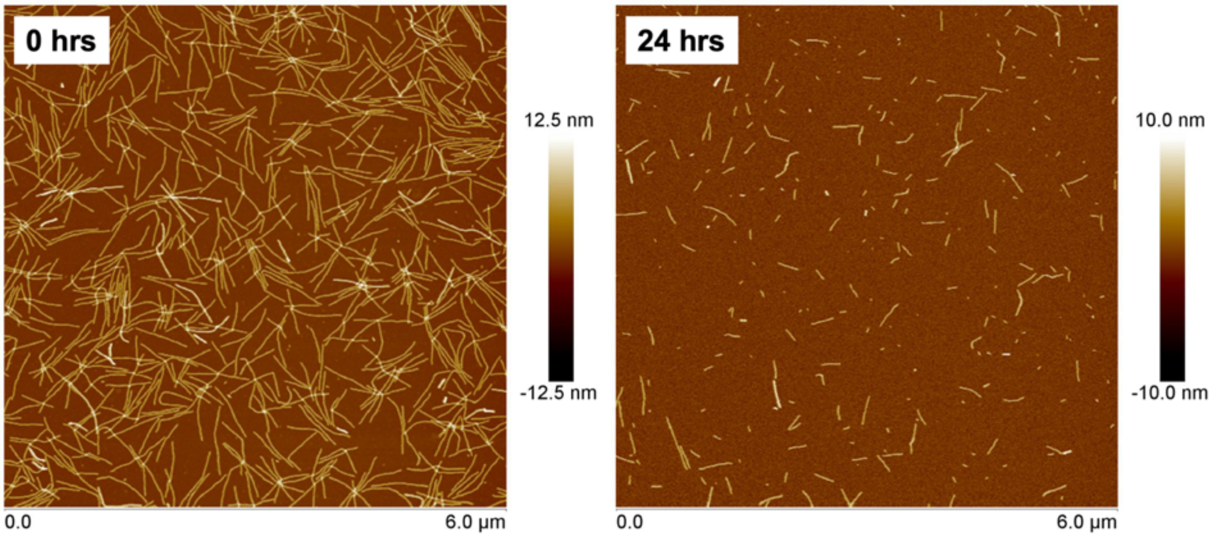

Supplementary Figure 31| Mixed bC8$_8$-Cy3-DNAa and bC8$_8$-Cy5-DNAa fibers (assembled together) 0 hours and 24 hours following mixing. AFM in air on mica. At 0 hours: L_N = 591 nm, D = 1.07 (N = 146); at 24 hours: L_N = 149 nm, D = 1.56 (N = 151). Image width: 6 µm.

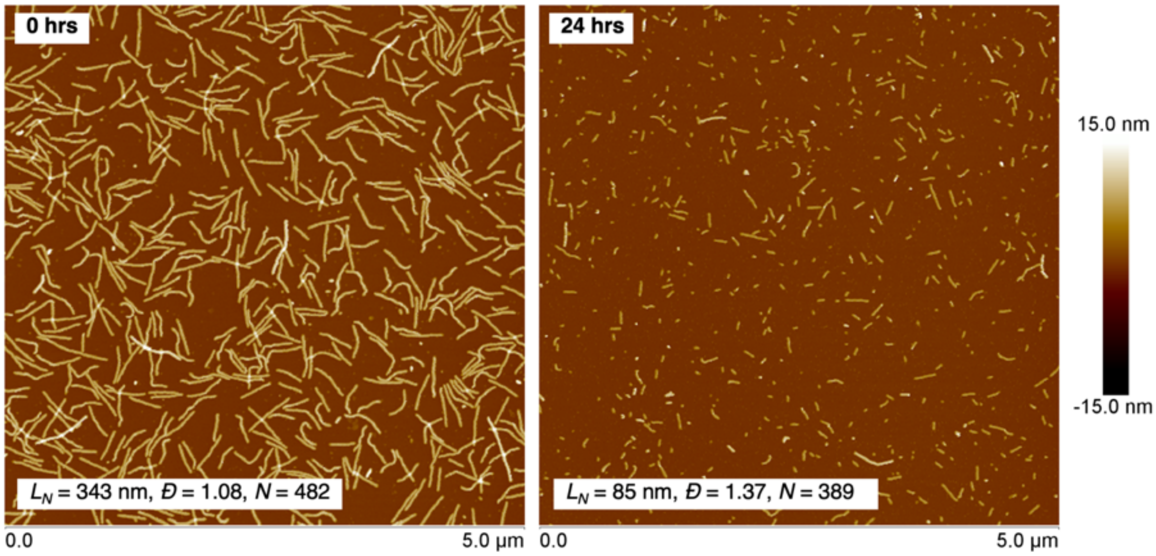

Supplementary Figure 32| bC8$_8$-DNAb fibers without melamine added at 0 hours and 24 hours following assembly. AFM in air on mica. Image width: 5 µm.

SI-II. Supplementary Experimental Procedures

II-a. General materials and DNA-oligomer sequences

Unless otherwise stated, all starting materials were purchased from commercial suppliers and used without further purification. Magnesium acetate tetrahydrate, triethylamine, N,N-diisopropylethylamine (DIPEA), tris(hydroxymethyl)-aminomethane (Tris), urea, EDTA, glycerol, 1,2-dodecanediol (cat. # 213721), 1,2-octanediol (cat. # 213705), tetrahydrofuran, dichloromethane (DCM), acetonitrile, ammonium persulfate (APS), and all other solvents were used as purchased from Sigma-Aldrich. 4,4′-Dimethoxytriphenylmethyl chloride (DMT-Cl, cat. # J90802) was purchased from AK Scientific. Acetic acid and boric acid were purchased from Fisher Scientific. GelRed™ nucleic acid stain was purchased from Biotium Inc. Concentrated ammonium hydroxide, acrylamide/bis-acrylamide (40% 19:1 solution) and TEMED were obtained from Bioshop Canada Inc. and used as supplied. 1 µmol Universal 1000Å LCAA-CPG supports and standard reagents used for automated DNA synthesis were purchased through Bioautomation. Cyanine 3 (cat. # 10-5913-95) and Cyanine 5 (cat. # 10-5915-95) phosphoramidites were purchased from Glen Research. N,N-Diisopropylamino cyanoethyl phosphonamidic-Cl (cat. # RN-1505) was purchased from ChemGenes. 10 X TAM g buffer consisted of 400 mM tris(hydroxymethyl)aminomethane and 125 mM magnesium acetate tetrahydrate at pH 8.0 (adjusted using acetic acid). TEAA buffer was made by adding 7 mL of triethylamine (TEA) to water before the adjusting the pH to 8.0 with glacial acetic acid and diluting to 1 L total. The extinction coefficients of each specific DNA-oligomer used were estimated by calculating the weighted averages of the extinction coefficients of the nucleotides in the sequences.

Sequences of DNA-oligomers used. Atypical amidite/nucleotide codes: (**D** = branched C12), (**O** = branched C8), **Cy3** = cyanine-3), (**Cy5** = cyanine 5).

**Name**	**Sequence (5’ to 3’)**
**$C12_{12}$-DNAa**	**DDDDDDDDDDDD**TTTTTCAGTTGACCATATA
**$C12_{10}$- DNAa**	**DDDDDDDDDD**TTTTTCAGTTGACCATATA
**$C12_{8}$- DNAa**	**DDDDDDDD**TTTTTCAGTTGACCATATA
**$C8_{12}$-DNAa**	**OOOOOOOOOOOO**TTTTTCAGTTGACCATATA
**$C8_{10}$-DNAa**	**OOOOOOOOOO**TTTTTCAGTTGACCATATA
**$C8_{8}$-DNAa**	**OOOOOOOO**TTTTTCAGTTGACCATATA
**$C8_{8}$-DNAb**	**OOOOOOOO**TTCTTGTTACAGCAGCATC
**$C8_{8}$-Cy3-DNAa**	**OOOOOOOO-Cy3-**TTTTTCAGTTGACCATATA
**$C8_{8}$-Cy5-DNAa**	**OOOOOOOO-Cy5-**TTTTTCAGTTGACCATATA

II-b. Instrumentation

Standard automated oligonucleotide solid-phase synthesis was performed on a Mermade MM6 Synthesizer from Bioautomation. HPLC purification was carried out on an Agilent Infinity 1260. DNA quantification measurements were performed by UV absorbance with a NanoDrop Lite spectrophotometer from Thermo Scientific. Thermal annealing of all nucleic acids was conducted using an Eppendorf Mastercycler® 96 well thermocycler. Liquid Chromatography Electrospray Ionization Mass Spectrometry (LC-ESI-MS) was carried out using Dionex Ultimate 3000 coupled to a Bruker MaXis Impact™ QTOF. Dynamic light scattering (DLS) experiments were carried out using a DynaProTM Instrument from Wyatt Technology. Transmission electron microscopy images were acquired using a Delong America LVEM5 5 kV Benchtop EM instrument. Atomic force microscopy was performed on a MultiMode 8 instrument from Bruker. ScanAsyst mode was used. Bruker ScanAsyst Air probes (2 nm nominal tip radius) were used for air imaging. Nuclear magnetic resonance (NMR) spectra were recorded on Bruker 400 MHz or 500 MHz 1H, 13C, and 31P. Fluorescence measurements were performed using a SpectraMax i3x Multi-Mode Microplate Reader. Variable temperature fluorescence experiments were carried out on a LightCycler 96 (Roche) instrument.

II-c. Synthesis of branched alkyl chain phosphoramidites

Branched C12 phosphoramidite

The branched C12 phosphoramidite was synthesized and purified as described by Juliano and Laing[1].

Branched C8 phosphoramidite

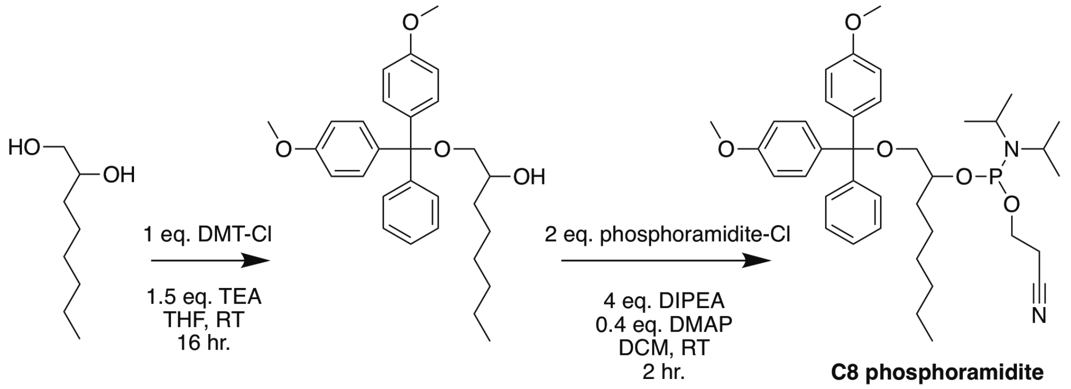

Synthetic route for branched C8 phosphoramidite

The mono-DMT protected C8 was synthesized using a procedure adapted from Skrzypczynski and Wayland[2]. Briefly, 1,2-octanediol was dissolved in tetrahydrofuran with 1.5 equivalents of triethylamine. 1 equivalent of 4,4'-dimethoxytrityl chloride was added in four parts over the course of two hours, and the reaction was then left at room temperature for 16 hours. Celite was added and the solvent was removed by rotary evaporation. Flash chromatography with solid loading was used to purify the mono-DMT protected product.

5. 1-(bis(4-methoxyphenyl)(phenyl)methoxy)octane-2-ol:

^{1}H NMR (500.3 MHz, $CDCl_3$) δ 7.46 (d, 2H, J = 6.9 Hz); 7.35 (d, 4H, J = 6.25 Hz); 7.30 (t, 2H, J = 7.3 Hz); 7.23 (t, 1H, J = 7.3 Hz); 6.85 (d, 4H, J = 8.0 Hz); 3.80 (s, 6H); 3.21-3.16 (m, 1H); 3.07-3.01 (m, 1H); 2.41-2.33 (m, 1H); 1.50-1.19 (m, 10H); 0.89 (t, 3H, J = 7.1 Hz).

1-(bis(4-methoxyphenyl)(phenyl)methoxy)octane-2-ol was dissolved in anhydrous DCM under inert gas. 4 equivalents of DIPEA and 0.4 eq. DMAP were added, followed by the slow addition of 2 equivalents of phosphoramidite chloride. The reaction was left at room temperature for 2 hours. The C8 phosphoramidite was purified by column chromatography using a mixture of Hex/EtOAC/TEA at 90/10/2 (v/v/v). Yield 82 %.

6. 1-(bis(4-methoxyphenyl)(phenyl)methoxy)octan-2-yl (2-cyanoethyl) diisopropylphosphoramidite

HRMS (ESI-QTOF) m/z: $[M + K]^+$ Calcd for $C_{38}H_{53}N_2O_5PK$ 687.3324; Found 687.3317. ^{1}H NMR (500.3 MHz, $CDCl_3$) δ 7.50-7.46 (m, 2H) ; 7.40-7.34 (m, 4H) ; 7.30 (q, 2H, J = 8.05 Hz) ; 7.23 (t, 1H, J = 7.9Hz) ; 6.86 (t, 4H, J = 8.9 Hz) ; 4.04-3.96 (m, 1H) ; 3.89-3.84 (m, 0.5H) ; 3.82 (s, 3H) ; 3.81 (s, 3H) ; 3.77-3.53 (m, 3H) ; 3.22-3.16 (m, 1H) ; 3.15-3.10 (m, 0.5H) ; 3.03-2.98 (m, 0.5H) ; 2.63 (t, 1H, J = 6.7 Hz) ; 2.45-2.38 (m, 1H) ; 1.83-1.72 (m, 0.5H) ; 1.71-1.51 (m, 2H) ; 1.39-1.14 (m, 17H) ; 1.09 (d, 3H, J = 6.4 Hz) ; 0.89 (t, 3H, J = 6.8 Hz).

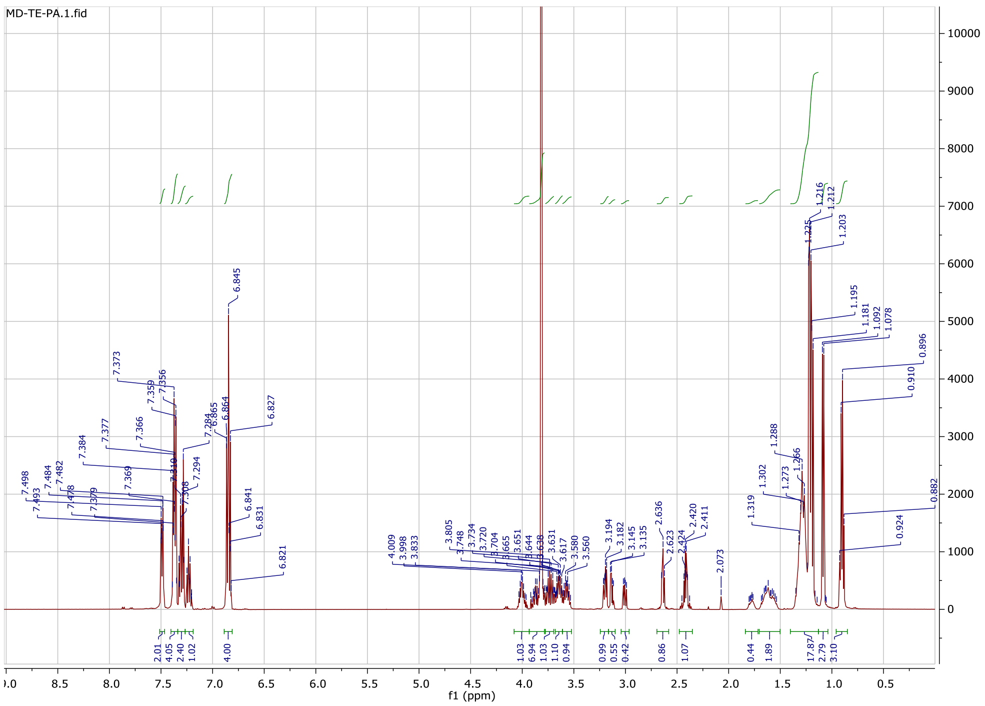

^{1}H NMR (500.3 MHz, $CDCl_3$) of 1-(bis(4-methoxyphenyl)(phenyl)methoxy)octan-2-yl (2-cyanoethyl) diisopropylphosphoramidite (6)

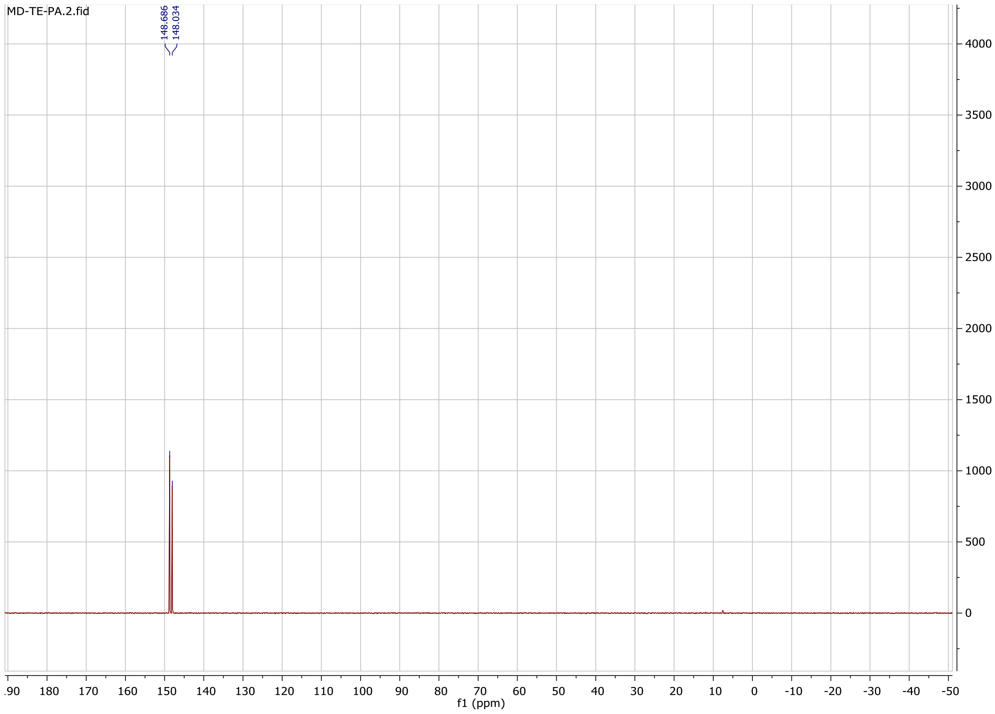

^{31}P NMR **(203 MHz, $CDCl_3$): δ (ppm) of 1-(bis(4-methoxyphenyl)(phenyl)methoxy)octan-2-yl (2-cyanoethyl) diisopropylphosphoramidite (6)**

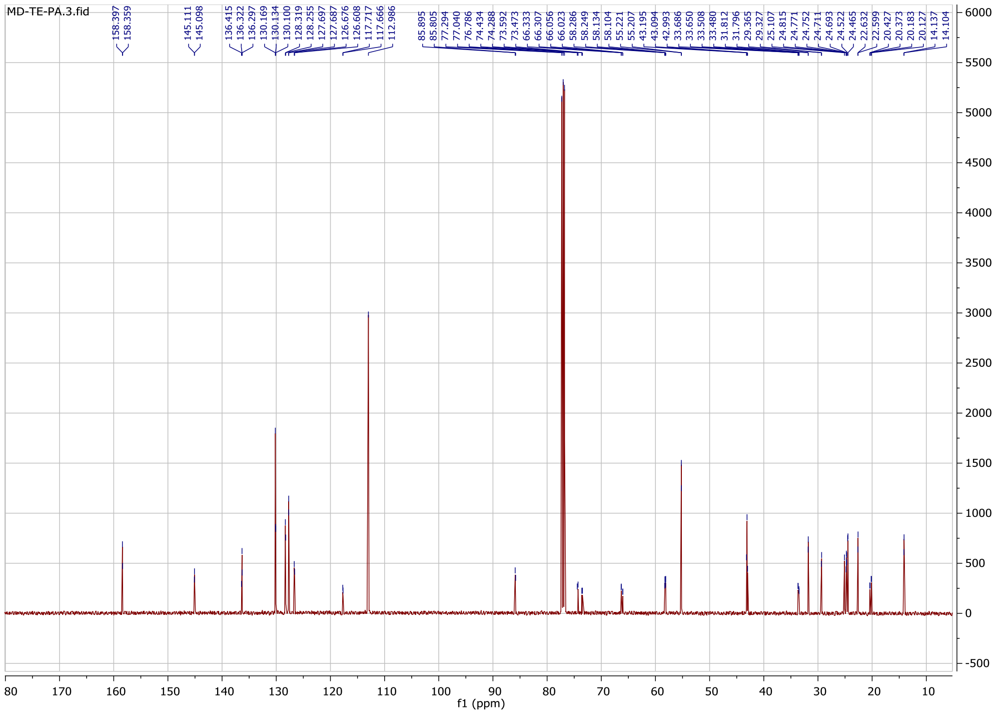

^{13}C NMR **(126 MHz, $CDCl_3$) of 1-(bis(4-methoxyphenyl)(phenyl)methoxy)octan-2-yl (2-cyanoethyl) diisopropylphosphoramidite (6)**

II-d. Solid-phase synthesis and purification of sequence-defined DNA-oligomers

All solid-phase syntheses were performed on a 1 µmol scale using universal 1000 Å LCAA-CPG solid-supports. Coupling efficiencies were monitored following removal of the dimethoxytrityl (DMT) 5'-OH protecting group.

Solid-phase synthesis of branched DNA-oligomers

Branched phosphoramidite monomers (C8 or C12) were dissolved in anhydrous acetonitrile at 0.1 mM and attached to the automated DNA synthesizer. The DNA blocks of all branched DNA-amphiphiles were synthesized first using standard solid-phase synthesis conditions (the hydrophobic block is always on the 5' end). Automated synthesis was then continued with the branched phosphoramidites directly on the DNA block attached to the solid support. An extended coupling time of 5 minutes was used for the branched monomers. Following synthesis, the strands were cleaved from the solid support and the bases deprotected by incubating in 30 % ammonium hydroxide in water for 24 hours at room temperature.

Purification of branched DNA-oligomers

The presence of the hydrophobic block on the 5' end of the oligomer allowed purification of the full-length strand by reverse-phase HPLC. 0.5 OD of crude oligomer in 20-100 µL of Millipore water was injected into a Hamilton PRP-1 5 µm 2.1x150 mm column at 60 °C. A gradient from 3 % to 70 % of acetonitrile in TEAA buffer was run over 30 minutes. Absorbance at 260 nm was monitored to detect DNA-amphiphiles. The most hydrophobic peak corresponded to the full-length product in all cases.

Summary of mass spectrometry characterization for branched DNA-amphiphiles

Oligomer	Expected mass	Found mass
**C12$_{8}$-DNAa**	7878.18	7878.13
**C12$_{10}$-DNAa**	8406.48	8406.41
**C8$_{8}$-DNAa**	7429.68	7429.65
**C8$_{10}$-DNAa**	7845.86	7845.78
**C8$_{12}$-DNAa**	8262.03	8261.91
**C8$_{8}$-Cy3-DNAa**	7936.92	7935.66
**C8$_{8}$-Cy5-DNAa**	7962.94	7961.33
**C8$_{8}$-DNAb**	7415.65	7415.11

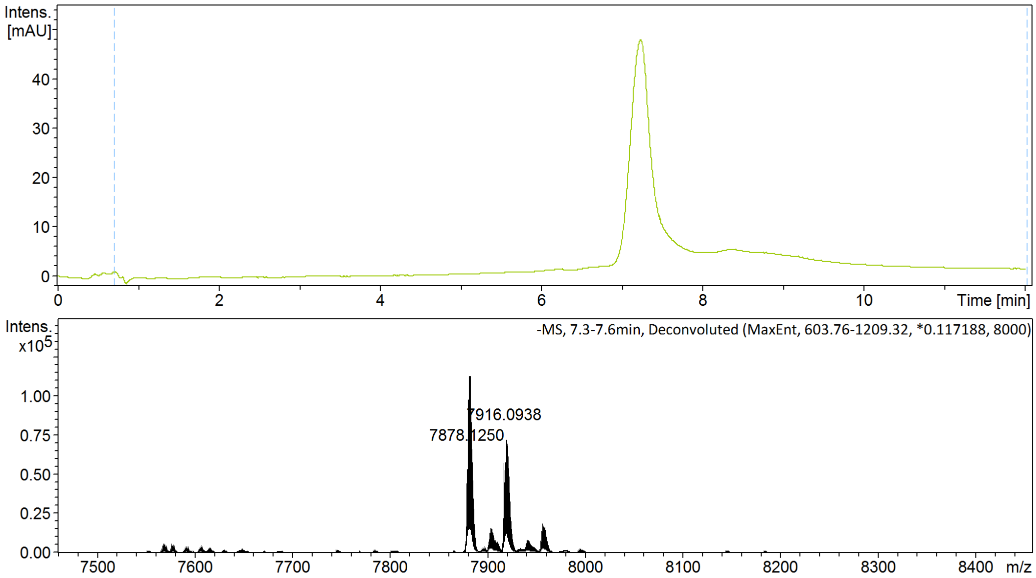

LC-MS of C12$_{8}$-DNAa. LC trace shows absorbance at 260 nm

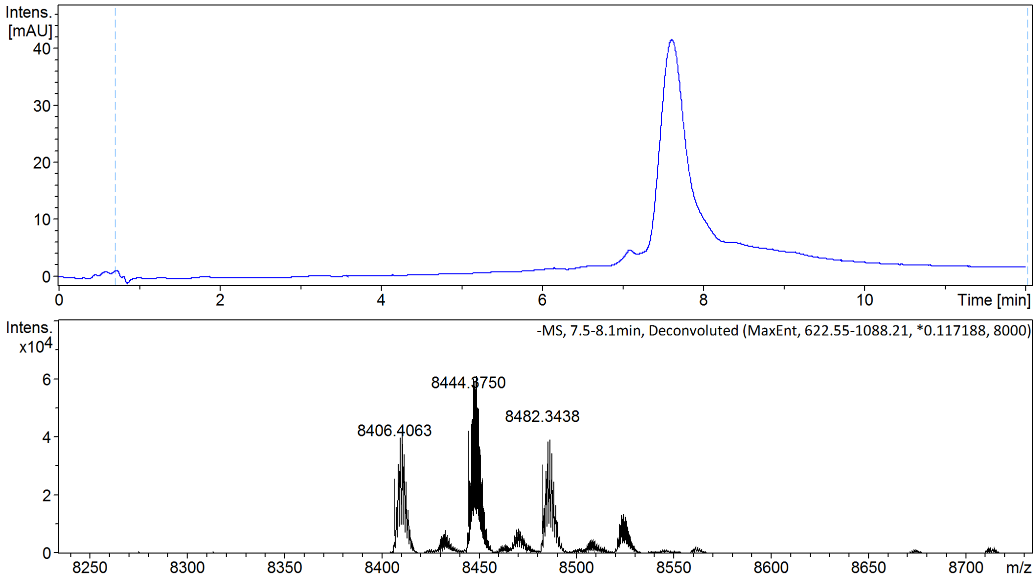

LC-MS of C12$_{10}$-DNAa. LC trace shows absorbance at 260 nm

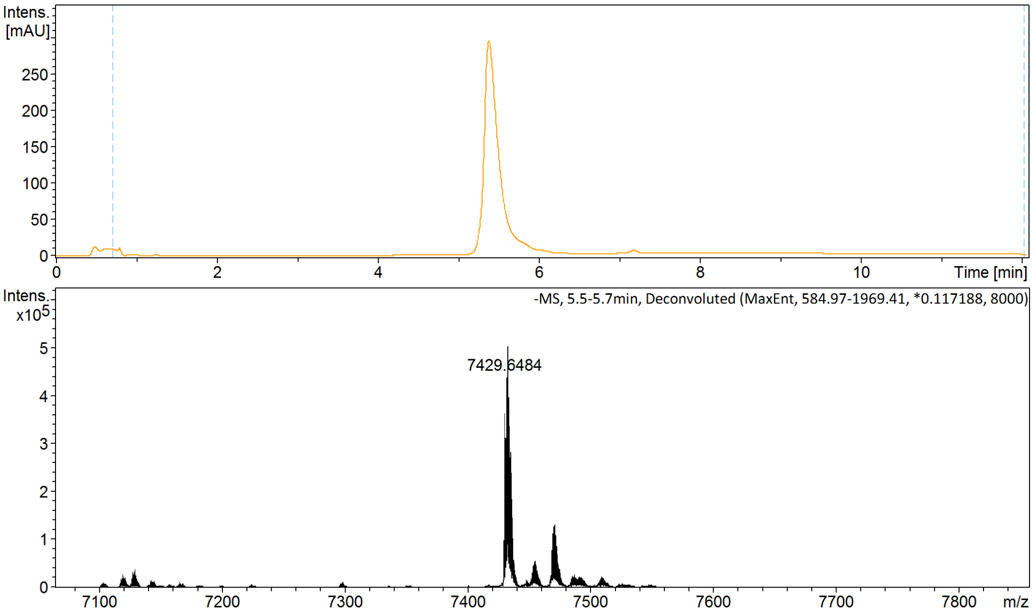

LC-MS of C8$_8$-DNAa. LC trace shows absorbance at 260 nm

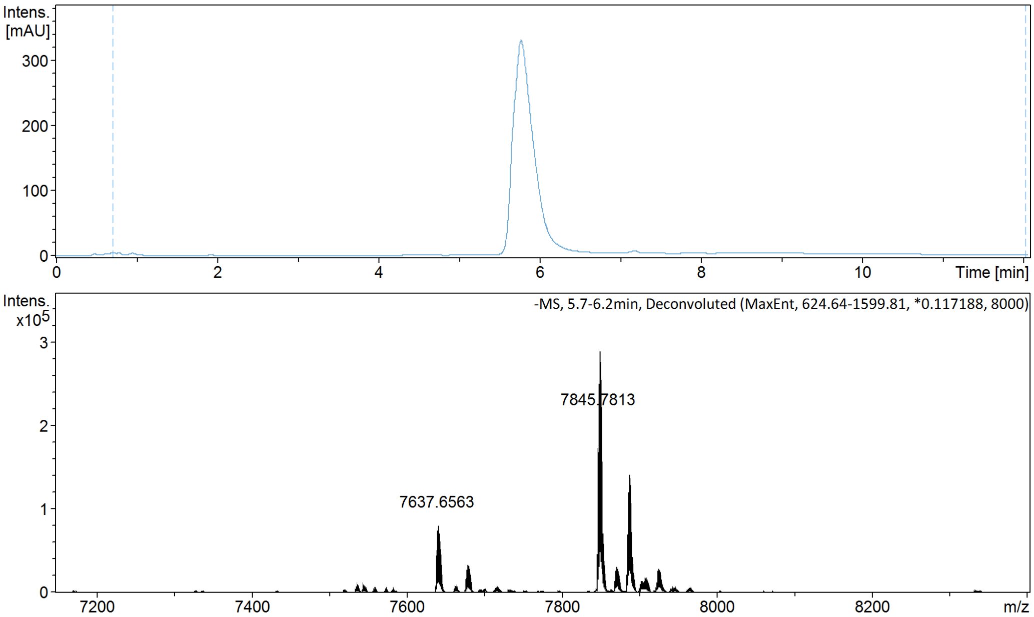

LC-MS of C8$_{10}$-DNAa. LC trace shows absorbance at 260 nm

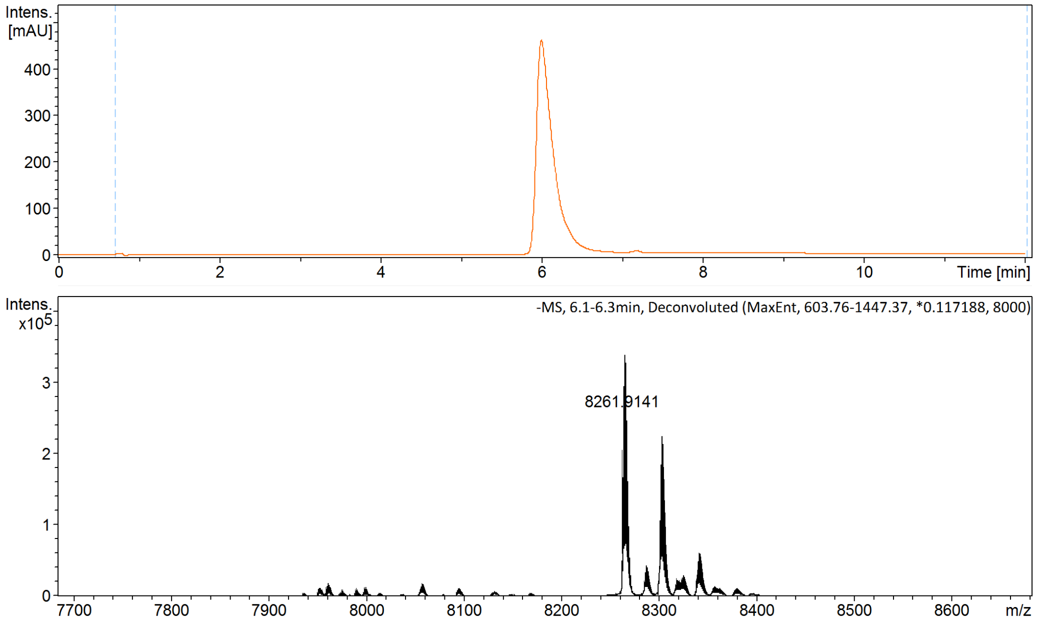

LC-MS of C8$_{12}$-DNAa. LC trace shows absorbance at 260 nm

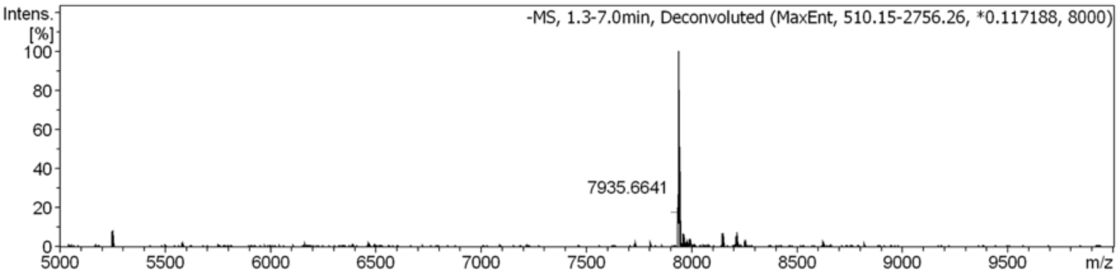

MS of C8$_8$-Cy3-DNAa

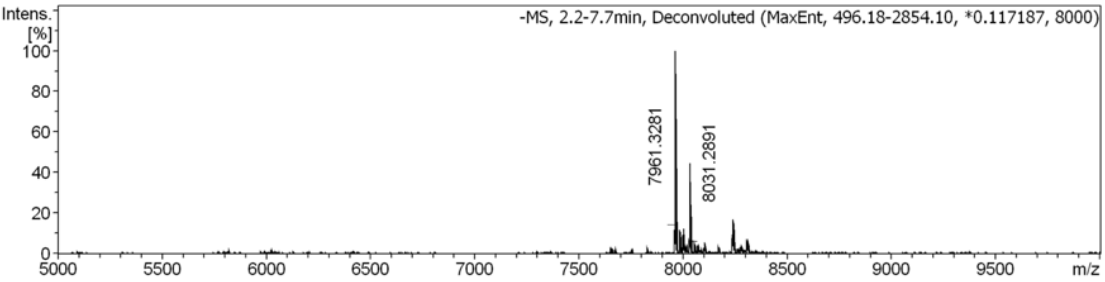

MS of C8$_8$-Cy5-DNAa

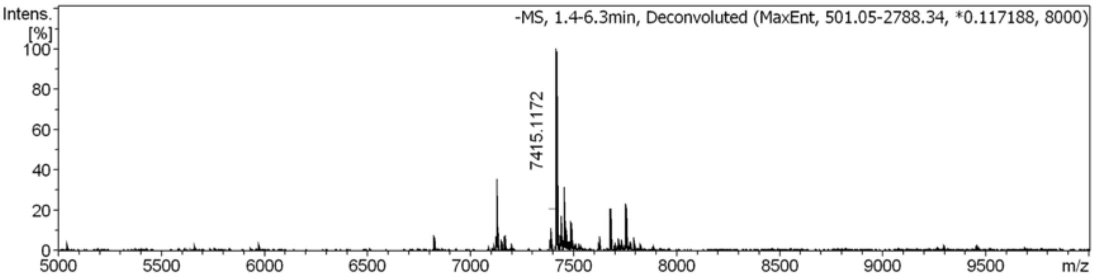

MS of C8$_8$-DNAb

II-e. Dynamic light scattering

15 μL of desired sample was assembled using stocks of buffer, ultrapure water, and DNA-oligomer that had been previously filtered using nylon centrifugal spin filters with a 200 nm cutoff. Assembled samples were analysed in a quartz cuvette with a minimum sample volume of 10 μL. Diffusion coefficient data was collected in triplicate from separate assembled samples. Displayed histograms are from one representative measurement. Each measurement consisted of 15 acquisitions, each ten seconds long.

II-f. Variable temperature assembly studies

*In situ fluorescence (**Figure 1, Supplementary Figure 5, 8, 11**)*
See methods section in main text.

*Fluorescence measured at room temperature (**Supplementary Figure 6, 7**)*
Samples were heated as described in the text or figure legends. Aliquots were directly taken from the sample at the desired temperatures and cooled to room temperature before measurement. 10 mol% **bC8$_8$-Cy3-DNAa** was used with **bC8$_8$-DNAa** for the fluorescence experiments.

II-g. Estimation of molecular geometry

Molecular lengths were estimated assuming an average bond length of 1.5 Å and bond angle of 109.5°. Lengths of bC8 and bC12 alkyl chains were calculated using only the branched segment. Disc radii were calculated assuming the backbone assumes a perfect circle in one plane while maintaining 109.5° bond angles.

II-h. Molecular dynamics simulations

Name	Description	Size (Å^3)	Number of atoms	Length
**Supplementary Movie 2_C88**	Single bC8$_8$-DNAa molecule in water with counterions simulated at room temperature	155x75x66	80013	300 ns
**Supplementary Movie 1_C88_85C**	Single bC8$_8$-DNAa molecule in water with counterions simulated at 85 °C	155x75x67	80013	300 ns
**Supplementary Movie 3_C810**	Single bC8$_{10}$-DNAa molecule in water with counterions simulated at room temperature	155x75x68	73587	300 ns

II-i. Seeded growth of length defined fibers

The number average length (L_N), weight average length (L_W), and the dispersity ($Đ$) was calculated as described below[3]:

(1) $$L_N = \frac{\sum_{i=1}^{n} N_i L_i}{\sum_{i=1}^{n} N_i}$$

(2) $$L_W = \frac{\sum_{i=1}^{n} N_i L_i^2}{\sum_{i=1}^{n} N_i L_i}$$

(3) $$Đ = \frac{L_W}{L_N}$$

Where L_i is the length of fiber i and N_i is the number of fibers i.

II-j. Förster resonance energy transfer (FRET) studies

FRET efficiency was calculated using the following equation:

(4) $$E = 1 - F'_D/F_D$$

Where E is FRET efficiency, F'_D is donor fluorescence in the presence of acceptor, and F_D is donor fluorescence in the absence of acceptor. In this system Cy3 was the donor and Cy5 was the acceptor. The “no FRET” control was used to determine F_D.

References

1 Laing, B. & Juliano, R. DNA Three-Way Junctions Stabilized by Hydrophobic Interactions for Creation of Functional Nanostructures. *Chembiochem* **16**, 1284-1287 (2015). https://doi.org:10.1002/cbic.201500034

2 Skrzypczynski, Z. & Wayland, S. New reagents for the introduction of reactive functional groups into chemically synthesized DNA probes. *Bioconjugate Chemistry* **14**, 642-652 (2003). https://doi.org:10.1021/bc025657j

3 Gilroy, J. *et al.* Monodisperse cylindrical micelles by crystallization-driven living self-assembly. *Nature Chemistry* **2**, 566-570 (2010). https://doi.org:10.1038/NCHEM.664
