# Supplementary material for: Heat-activated growth of metastable and length-defined DNA fibers expands traditional polymer assembly
Source: Nat Commun. 2024 May 23;15:4384. doi: 10.1038/s41467-024-48722-2 (PMC11116425; doi:10.1038/s41467-024-48722-2)
Supplement: Supplementary file 3 — Description of Additional Supplementary Files [file 41467_2024_48722_MOESM3_ESM.pdf]

Description of Additional Supplementary Files

Supplementary Movie 1:
Single $bC8_8$-DNAa molecule in water with counterions simulated at 85 °C.

Supplementary Movie 2:
Single $bC8_8$-DNAa molecule in water with counterions simulated at room temperature

Supplementary Movie 3:
Single $bC8_{10}$-DNAa molecule in water with counterions simulated at room temperature
